# CXCR7 promotes melanoma tumorigenesis via Src kinase signaling

**DOI:** 10.1038/s41419-019-1442-3

**Published:** 2019-02-25

**Authors:** Siran Xu, Jiaze Tang, Chunying Wang, Jie Liu, Yan Fu, Yongzhang Luo

**Affiliations:** 1 Peking University-Tsinghua University-National Institute of Biological Sciences Joint Graduate Program (PTN), School of Life Sciences, Peking University, Beijing, China; 2 The National Engineering Laboratory for Anti-Tumor Protein Therapeutics, Tsinghua University, Beijing, China; 3 Beijing Key Laboratory for Protein Therapeutics, Tsinghua University, Beijing, China; 4 Cancer Biology Laboratory, School of Life Sciences, Tsinghua University, Beijing, China

## Abstract

Chemokine receptors have been documented to exert critical functions in melanoma progression. However, current drugs targeting these receptors have limited efficacy in clinical applications, suggesting the urgency to further explore the roles of chemokine receptors in melanoma. Here we found that C–X–C chemokine receptor 7 (CXCR7) was the most highly expressed chemokine receptor in murine melanoma cell lines. In addition, the expression level of CXCR7 was positively correlated with melanoma progression in the clinical samples. High CXCR7 expression was associated with shorter overall survival in melanoma patients. Increased expression of CXCR7 augmented melanoma proliferation in vitro and tumor growth in vivo, whereas knockout of CXCR7 exhibited significant inhibitory effects. Moreover, our data elucidated that CXCR7 activated Src kinase phosphorylation in a β-arrestin2-dependent manner. The administration of the Src kinase inhibitor PP1 or siRNA specific for β-arrestin2 abolished CXCR7-promoted cell proliferation. Importantly, CXCR7 also regulated melanoma angiogenesis and the secretion of vascular endothelial growth factor (VEGF). Subsequent investigations revealed a novel event that the activation of the CXCR7-Src axis stimulated the phosphorylation of eukaryotic translation initiation factor 4E (eIF4E) to accelerate the translation of hypoxia-inducible factor 1α (HIF-1α), which enhanced the secretion of VEGF from melanoma cells. Collectively, our results illuminate the crucial roles of CXCR7 in melanoma tumorigenesis, and indicate the potential of targeting CXCR7 as new therapeutic strategies for melanoma treatment.

## Introduction

Melanoma is one of the most prevalent and lethal human malignancies in Western countries, with a markedly rising incidence for over three decades^1,2^. While novel clinical therapeutics, such as *BRAF*-targeted and immune therapies, have improved patients’ outcomes in recent years^3^, malignant melanoma is still the most deadly skin cancer. Thus, it is urgent to gain further insights into the mechanisms underlying melanoma progression and to develop new therapeutic strategies.

Chemokine receptors are a subgroup of G protein-coupled receptors that can activate various downstream signaling pathways after stimulated by cognate chemokines^4,5^. Aside from their originally characterized functions in leukocyte trafficking^6^, chemokine receptors have been identified on most types of cells and participate in a large variety of physiological and pathological processes^7^. During the last decade, mounting evidences have demonstrated the pivotal contributions of chemokine receptors to melanoma progression^8–11^. Overexpression of CXCR1 and CXCR2 enhanced melanoma cell proliferation and tumorigenesis^12^. Antagonists targeting these two receptors hindered melanoma growth by inhibiting angiogenesis, cell proliferation, and survival^13^. In addition, CCR7 and CXCR4 have been shown to govern melanoma lymph node and pulmonary metastases, respectively^14,15^. Based on these findings, a number of specific antagonists and antibodies have been developed. However, the outcomes remain limited in preclinical models and clinical trials, suggesting that further investigations about the biological significance of chemokine receptors in melanoma are required^11,16^.

CXCR7, originally recognized as an orphan receptor^17^, has been deorphanized as a chemokine receptor interacting with CXCL11 and CXCL12^18,19^. It has a 10-fold higher binding affinity for CXCL12 than the canonical receptor CXCR4^18^. Although recently renamed as atypical chemokine receptor 3 (ACKR3) due to the defects in initiating classical G protein-coupled receptor pathways^19,20^, CXCR7 has been reported to activate downstream signalings through β-arrestins, rather than G-protein complexes^21,22^. The prominent roles of CXCR7 in developmental and physiological contexts have been documented by several studies^23,24^. More importantly, emerging evidences have demonstrated that CXCR7 expression is elevated in a broad range of malignant tumors and exhibits diverse functions in tumor progression. The upregulation of CXCR7 confers growth advantages to breast and lung cancer^19,25^. CXCR7 also promotes tumor growth and angiogenesis by supporting VEGF secretion in hepatocellular carcinoma^26,27^. In prostate cancer, the level of CXCR7 is regulated by CXCR4, IL8, and androgen receptor, and is associated with cell adhesion, proliferation, survival, and angiogenesis^28–30^. In addition, CXCR7 has been detected by immunohistochemistry staining of tissue samples from melanoma patients^31^. To date, however, few studies have reported the biological functions and molecular mechanisms of CXCR7 in melanoma^32,33^.

In the present study, our results showed that CXCR7 was highly expressed in melanoma cell lines and its level in patient samples was positively correlated with melanoma progression. In addition, high CXCR7 expression predicted worse overall survival in melanoma patients. Subsequent investigations demonstrated that CXCR7 augmented melanoma proliferation through β-arrestin2-mediated activation of Src kinase. Moreover, CXCR7 contributed to angiogenesis of melanoma. The CXCR7-Src axis enhanced eIF4E phosphorylation to fuel HIF-1α translation, which supported VEGF secretion. Our findings shed light on the critical roles of CXCR7 in melanoma tumorigenesis, and suggest that targeted intervention of CXCR7 may provide therapeutic benefits for melanoma patients.

## Materials and methods

### Cell culture, reagents, and antibodies

Murine melanoma cell lines B16-F0, B16-F1, B16-F10, and human melanoma cell line A375 were purchased from the American Type Culture Collection (Manassas, VA, USA). HEK-293T, 4T1 and A549 cells were from the China Infrastructure of Cell Line Resources (Beijing, China). All cell lines were cultured in Dulbecco’s Modified Eagle’s Medium (Hyclone, Logan, UT, USA) with 10% fetal bovine serum (Gibco BRL, Grand Island, NY, USA), 100 U/ml penicillin and 100 μg/ml streptomycin sulfate (Hyclone). The cells were cultured at 37 °C with 5% CO_2_. Recombinant murine and human CXCL12 were purchased from Peprotech (Rocky Hill, NJ, USA). PP1, U0126, AMD3100, AG1478, and MG132 were from Selleck Chemicals (Houston, TX, USA). MNK1/2 inhibitor cercosporamide was purchased from R&D Systems (Minneapolis, MN, USA). Antibodies against p-AKT, AKT, p-Src, Src, p-S6K, S6K, p-ERK, ERK, p-4E-BP1, 4E-BP1, p-eIF4E, eIF4E, and β-arrestin2 were from Cell Signaling Technology (Danvers, MA, USA). Antibodies against HIF-1α, VEGF, and β-arrestin1 were from Abcam (Shanghai, China). Antibody against CD31 were from BD Biosciences (San Jose, CA, USA). Antibody against EGFR was from Santa Cruz Biotechenology (Dallas, TX, USA). Antibodies against CXCR7, β-actin and VHL were purchased from GeneTex (Irvine, CA, USA). Horseradish peroxidase (HRP)-conjugated goat anti-mouse or anti-rabbit immunoglobulin G (IgG) antibodies were from Abcam. Fluorescein isothiocyanate (FITC)-conjugated goat anti-rat IgG antibody was from ZSGB-BIO (Beijing, China).

### Quantitative RT-PCR

The total RNA were isolated from cells and the gene expression levels were determined by quantitative RT-PCR (qRT-PCR) assays as previously described^34^. In brief, the total RNA were isolated by TRIZOL Reagent (Invitrogen, Thermo Fisher Scientific, Wilmington, DE, USA) and the synthesis of cDNA were performed by the First Strand cDNA Synthesis Kit (Fermentas, Thermo Fisher Scientific). The gene expressions were measured on the Mx3000P system (Stratagene, La Jolla, CA, USA) with specific primers. The sequence information for all the primers in the present study are listed in Supplementary Table 1. Relative quantitation of the gene expression was normalized by β-actin mRNA level following the 2^–ΔΔCt^ method and relative to the control group. Independent experiments were conducted in triplicate.

### Western blot

Cell lysate samples were collected from cells cultured in plates and the levels of indicated proteins were measured by western blot assays as previously described^34^. In brief, samples were subjected to SDS-PAGE and transferred to polyvinylidene difluoride membranes (Millipore, Billerica, MA, USA). The membranes were blocked with 5% fat-free milk and incubated with specific primary antibodies at 4 °C overnight. Then the membranes were incubated with HRP-conjugated secondary antibodies. The expressions of proteins were detected with an enhanced chemiluminescence system (Thermo Fisher Scientific). The detailed information about the antibodies used in western blot analysis are described in Supplementary Table 2.

### Immunohistochemistry and immunofluorescent staining

The paraformaldehyde-fixed, paraffin-embedded tumor tissues were cut into 6-μm sections. The sections were dewaxed, boiled in sodium citrate buffer solution (pH 6.0), and chilled to room temperature. The sections were then blocked with 10% goat serum and were incubated at 4 °C overnight with antibodies against CXCR7 (1:80, MAB42273, R&D Systems; 1:100, GTX100027, GeneTex), VEGF (1:100, ab1316, Abcam), ki67 (1:200, ab16667, Abcam), HIF-1α (1:200, ab1, Abcam), and CD31 (1:100, 550274, BD Biosciences). After washing twice with phosphate buffer solution (PBS, pH 7.0), the sections for immunohistochemistry staining were incubated with HRP-conjugated secondary antibodies. The protein expressions were visualized by incubating the sections with 3,3′-diaminobenzidine and the nuclei were counter-stained with hematoxylin. The immunohistochemistry images were obtained with Olympus IX71 optical microscope and the staining intensities were analyzed with Image-Pro Plus 6.0 (Media Cybernetics). The sections for CD31 immunofluorescent staining were incubated with FITC-conjugated secondary antibodies (1:200, ZF-0315, ZSGB-BIO) and the nuclei were stained by 2-(4-amidinophenyl)-6-indolecarbamidine dihydrochloride (DAPI). The immunofluorescent images were captured with Nikon A1 laser scanning confocal microscope (Plan Apo 40×/0.65 objectives) and analyzed with NIS-Elements AR 3.0 software (Nikon, Tokyo, Japan). All the images for quantification were captured under the same camera settings. Tumor angiogenesis was quantified as the ratios between CD31-positivie areas and DAPI-positive areas.

### Tissue microarray

Melanoma patient tissue microarray (ME1004) was purchased from Alenabio (Xi’an, China). The microarray contained 100 clinical specimens (median age 51 years, range 0.5–88, 51 males and 49 females), including 24 benign tumors, 56 primary malignant melanomas, and 20 metastatic malignant melanomas. The immunohistochemistry staining was performed with primary anti-CXCR7 antibody (R&D). CXCR7 expressions were determined by the staining intensities. Detailed information about the patients are listed in Supplementary Table 3.

### CRISPR-Cas9 mediated CXCR7 depletion

CXCR7-depleted B16-F10 and A375 cells were established by the CRISPR-Cas9 system according to the previous study^35^. Paired single-guide RNAs (sgRNAs) targeting the coding sequence of murine and human CXCR7 were designed by the online CRISPR Design tool (http://crispr.mit.edu/) and cloned into a Cas9-coding plasmid pSpCas9(BB)-2A-GFP (PX458, Addgene, Cambrige, UK). PX458 plasmid without sgRNA was used to construct wild-type control cells. Reparation of the two double-strand breaks guided by a pair of sgRNAs could introduce a targeted deletion. The constructed plasmids were transfected into cells by jetPRIME Transfection Reagent (Polyplus transfection, NY, USA) following the manufacturers’ instructions. After 48 h, GFP-positive cells were sorted by FACS and seeded as single cell into 96-well plates for clonal expansion. The genomic DNA of established clones were isolated by EasyPure Genomic DNA Kit (TransGen Biotech, Beijing, China). The knockout efficiencies were determined by amplification of genomic DNA with primers flanking the region deleted by a pair of sgRNAs. Detailed information about sgRNA sequences and genomic DNA amplification primers are listed in Supplementary Table 4.

### Lentivirus infection

Lentivirus infection was performed to construct CXCR7-overexpressing B16-F0 cells as previously described^36^. The packaging vector psPAX2, the envelope plasmid pVSVG, and the transfer plasmid pLentiCMV containing *Ackr3*-coding sequence were co-transfected into HEK-293T cells. After 72 h, the supernatant medium from HEK-293T was collected, filtered by an 0.45 μM filter, and added into B16-F0 cells. The medium was replaced with fresh culture medium after 48 h and the stably transfected cells were selected by medium containing puromycin (10 μg/ml).

### Cell proliferation assay

The cells were seeded into 96-well plates (1×10^4^ cells per well) and cell proliferations were measured after 24 h and 48 h, using the Cell Counting Kit-8 (CCK-8, Dojindo, Tokyo, Japan). According to the instructions, the culture mediums of various cells were replaced with 100 μl serum-free medium containing 10 μl CCK-8 solution. Then the cells were cultured at 37 °C and the proliferative rates were determined by measuring the absorbance at 450 nm wavelength using Varioskan Flash (Thermo Fisher Scientific). Independent experiments were repeated in triplicate.

### Overall survival analysis of melanoma patients

The publicly available pathology atlas of melanoma transcriptome^37^ were utilized to analyze the correlation between CXCR7 expression and overall survival in melanoma patients. The Kaplan–Meier survival analysis was performed by GraphPad. (https://www.proteinatlas.org/ENSG00000144476-ACKR3/pathology/tissue/melanoma).

### Colony formation assay

Cells were seeded into 6-well plates in triplicates at a density of 800 cells per well. After incubation for 12 days, colonies were fixed in 4% paraformaldehyde and stained with crystal violet. Then each well was washed with PBS twice. The number of individual colonies larger than 100 μm in diameter was counted using an inverted microscope (IX71; Olympus, Tokyo, Japan).

### Cell apoptosis analysis

Cells were seeded into 6-well plates and the rates of apoptosis were measured by an Annexin V-FITC/PI Apoptosis Detection Kit (CWbiotech, Beijing, China). According to the manufacturer’s instructions, cells were harvested and washed with cold PBS twice. After being resuspended in 100 μl of binding buffer, cells were incubated with FITC-Annexin V and propidium iodide (PI) for 15 min in the dark. Then flow cytometry analysis of apoptosis was performed using BD FACS Calibur (BD Biosciences, San Jose, CA, USA). The results were analyzed with FlowJo (FlowJo, LLC, Ashland, OR, USA).

### Small interfering RNA transfection

Transient downregulation of proteins was performed by small interfering RNA (siRNA) transfection. siRNAs targeting specific genes and scramble control siRNA were purchased from GenePharma (Shanghai, China). Cell transfections were performed with jetPRIME Transfection Reagent according to the instructions and the knockdown efficiencies were determined by western blot. The most effective sequences for all siRNAs used in the present study are listed in Supplementary Table 5.

### ELISA assay

VEGF secretion in cell culture supernates were measured by the enzyme-linked immunosorbent assay (ELISA). The culture mediums of various cells were replaced with serum-free medium at ~60% confluence. After 16 h, the conditioned mediums were collected and the secretion levels of VEGF were determined by murine or human VEGF ELISA kits (Dakewe Biotech Co., Ltd, Shenzhen, China) according to the manufacturer’s instructions. CXCL12 secretion were measured by murine CXCL12 ELISA kits (ab100741, Abcam, Shanghai, China) according to the manufacturer’s instructions. The results were measured at 450 nm with an ELISA reader (Varioskan Flash, Thermo Fisher Scientific). All the experiments were independently repeated three times.

### Animal study

Animal studies were approved by the Institutional Animal Care and Use Committees of Tsinghua University (Approval number: 14-LYZ3). To establish an orthotopic melanoma model, the constructed murine melanoma cells (5×10^5^ per mouse) were pre-mixed with Matrigel (Corning, New York, USA) and then subcutaneously inoculated into the right flank of female C57BL/6 mice (6-week old). Human melanoma cells (1×10^6^ per mouse) were subcutaneously inoculated into female BALB/c nude mice (6-week old). After the primary tumors formed, tumor volumes were monitored daily and calculated based on the formula: volume = 0.5ab^2^ (“a” is the long diameter of the tumor and “b” is the short diameter). After the mice were killed, tumor tissues were dissected, weighted, and fixed in 4% paraformaldehyde before embedded in paraffin.

### Statistical methods

Numerical data were presented as mean ± standard deviation (SD) except tumor volume data which were expressed as mean ± standard error of the mean (SEM). Statistical significance of differences between two groups were determined by the two-tailed, unpaired Student’s *t*-tests using GraphPad Prism (GraphPad Software, San Diego, CA, USA). The *χ*2 test was used to assess the correlation between protein expression and clinicopathologic stages. Overall survival analysis was assessed by log-rank test. A value of *p* < 0.05 was considered to be significant.

## Results

### CXCR7 is highly expressed in melanoma cells and positively correlated with melanoma progression

**Fig. 1:**
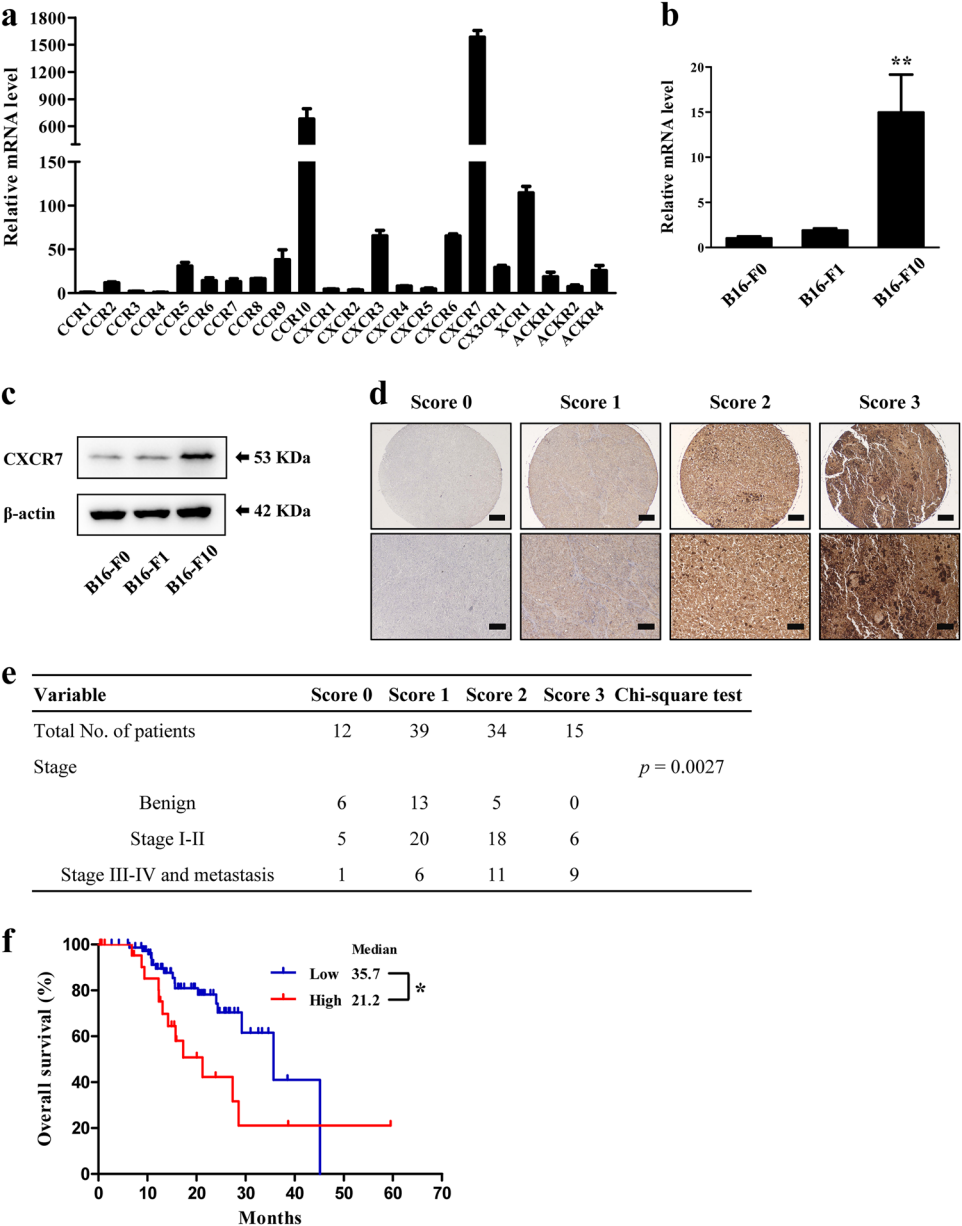
The expression of CXCR7 in metastatic melanoma cells and clinical samples. **a** The expression levels of chemokine receptors in B16-F10 cells were evaluated by qRT-PCR and normalized to *Ccr1* mRNA level. **b**, **c** The relative mRNA (**b**) and protein (**c**) levels of CXCR7 in B16-F0, B16-F1, and B16-F10 cells. The mRNA levels were normalized to B16-F0 cells. **d** Representative images of CXCR7 expression in benign, malignant, and metastatic melanoma samples that illustrate scores of 0, 1, 2, and 3. The top images were taken at ×100 original magnification (scale bar = 200 μm) and the bottom images were taken at ×200 original magnification (scale bar = 100 μm). **e** The correlation of CXCR7 staining scores with tumor stages. The *χ*2 test was used to assess the correlation between categorical variables. **f** Overall survival of melanoma patients with high (*n* = 24) or low (*n* = 78) CXCR7 expression. The expression cutoff = 3.51 FPKM. Overall survival was analyzed by Kaplan–Meier survival analysis and the log-rank test. The qRT-PCR experiments were independently repeated three times. Data are presented as mean ± SD; *p < 0.05, **p < 0.01 compared with B16-F0 and B16-F1 cells

To investigate the biological functions of chemokine receptors in melanoma, we evaluated the mRNA levels of chemokine receptors in murine melanoma cell lines by qRT-PCR. As shown in Fig. 1a, of all the receptors, CXCR7 was the most highly expressed one in B16-F10 cells. Similar results were observed in B16-F0 and B16-F1 cells (Figure S1). Intriguingly, highly metastatic B16-F10 cells expressed a remarkably higher level of CXCR7 than cells with lower malignancy (Fig. 1b, c), suggesting that CXCR7 is correlated with melanoma aggressiveness. To further verify this statement, we performed immunohistochemistry staining of CXCR7 on a patient tissue microarray and the tissues were scored according to the staining intensities (Fig. 1d). As shown in Fig.  1e, *χ*^2^test determined that the expression level of CXCR7 was significantly correlated with melanoma progression in the clinical samples (*p* = 0.0027). Moreover, we used a publicly available database (www.proteinatlas.org/pathology) to examine the clinical significance of CXCR7 expression as a prognostic marker for melanoma. The patients were classified into high-expression and low-expression groups based on CXCR7 levels. Kaplan–Meier survival analysis revealed that melanoma patients with high CXCR7 expression levels displayed significantly shorter overall survival (Fig. 1f).

### CXCR7 modulates melanoma cell proliferation in vitro and tumor growth in vivo

**Fig. 2:**
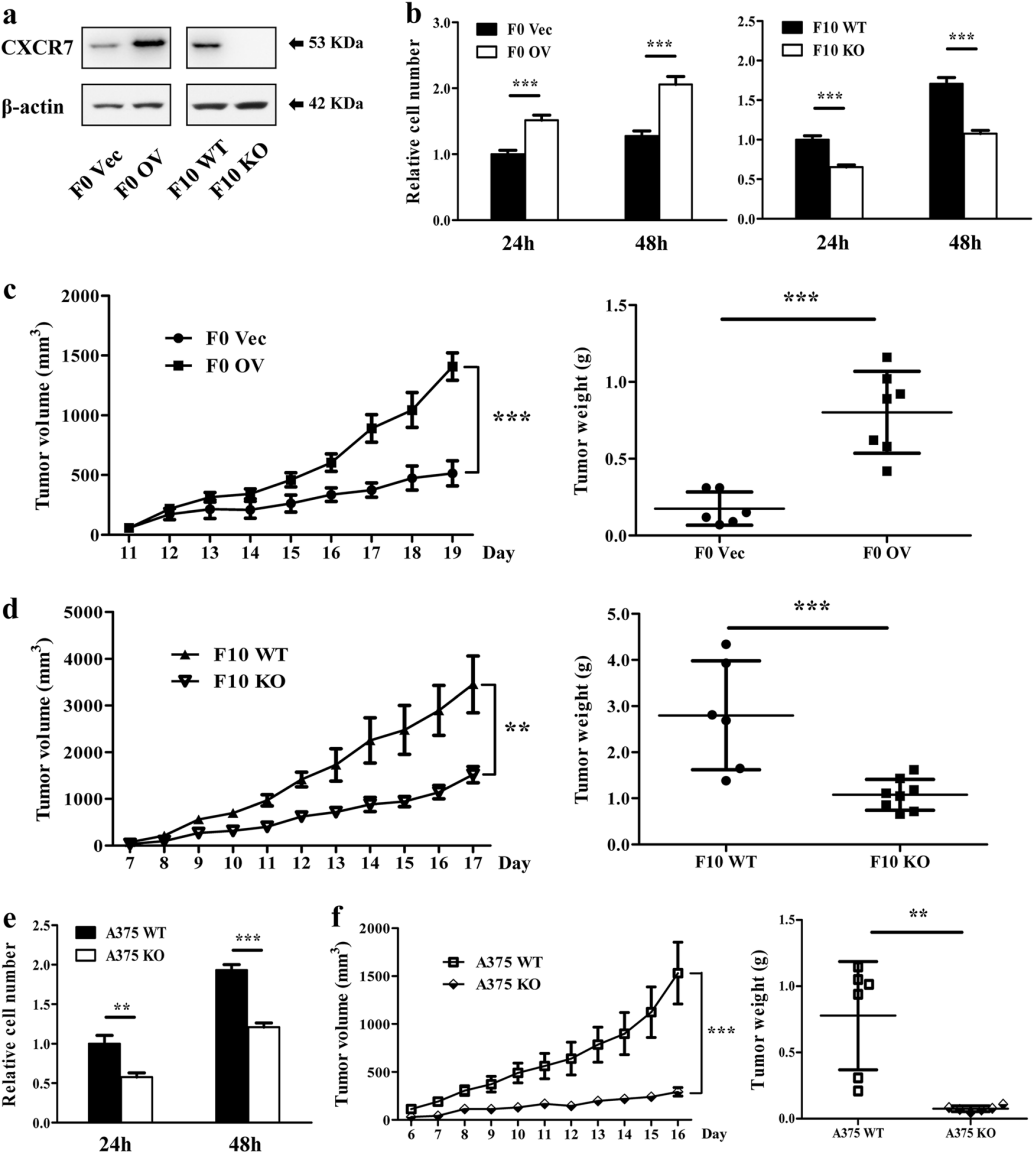
CXCR7 facilitates melanoma cell proliferation in vitro and tumor growth in vivo. **a** CXCR7 overexpression and depletion in B16-F0 cells and B16-F10 cells. **b** The effects of CXCR7 overexpression and depletion on melanoma cell proliferation in vitro. F0 Vec, F0 OV, and F10 WT, F10 KO cells were seeded into 96-well plates and cell proliferations were examined by CCK-8 assays after 24 h and 48 h. The proliferation rates were normalized to F0 Vec cells (left) or F10 WT cells (right) at 24 h. **c**, **d** The effects of CXCR7 overexpression (**c**) and depletion (**d**) on melanoma tumor volume and tumor weight in vivo (*n* = 6–8 for each group). **e**, **f** The effects of CXCR7 depletion on A375 cell proliferation in vitro (**e**) and tumor growth in vivo (**f**; *n* = 6 for each group). The proliferation rates were normalized to A357 WT cells at 24 h. Proliferation analyses were independently repeated three times. Tumor volumes are shown as mean±SEM, and other data are presented as mean ± SD; **p < 0.01, ***p < 0.001

Early studies reported that CXCR7 facilitates tumorigenesis in various types of cancer, but its functions in melanoma remain poorly characterized. Prompted by above findings, we sought to determine whether CXCR7 has functional roles in melanoma proliferation and tumor growth. To this end, B16-F0 cells overexpressing CXCR7 (F0 OV) or control vectors (F0 Vec) were constructed by stable transfection with lentivirus. On the other hand, we utilized CRISPR-Cas9 system to establish CXCR7-depleted B16-F10 cells (F10 KO) and wild-type controls (F10 WT). The manipulated expression of CXCR7 was validated by Western blot and genomic DNA amplification (Fig. 2a, S2a, S2b). Notably, CXCR7 alterations had no impact on the secretion of CXCL12 from melanoma cells (Figure S2c). As shown in Fig. 2b, cell proliferation in vitro was enhanced by overexpression of CXCR7, whereas loss of CXCR7 in B16-F10 cells suppressed proliferation in comparison with the controls. To characterize the roles of CXCR7 on melanoma growth in vivo, we subcutaneously implanted the constructed cell lines into mice and monitored tumor volumes. The overexpression or depletion efficiency in each group was confirmed by immunohistochemistry staining (Figure S2d). In the context of CXCR7 overexpression, F0 OV cells gave rise to larger tumors than the F0 Vec group, accompanied by a remarkable increase in tumor weight (Fig. 2c). As indicated by Ki67 staining, the F0 OV tumors were more proliferative than those derived from F0 Vec cells (Figure S2e). Consistently, F10 KO cells exhibited pronounced reductions in both tumor size and weight (Fig. 2d). The proliferative activity of the tumors was significantly suppressed by CXCR7 depletion (Figure S2e).

To further confirm these findings in human melanoma, we established CXCR7-depleted and control A375 cells (A375 KO and A375 WT), and verified the knockout efficiency in vitro and in vivo (Figure S2f–S2h). In congruence with the foregoing data, CXCR7 depletion inhibited human melanoma cell proliferation and tumor growth (Fig. 2e, f and S2h). Moreover, the administration of a blocking antibody against CXCR7 attenuated A375 WT tumor growth in vivo (Figure S2i). Based on these observations, we demonstrate that CXCR7 augments melanoma tumorigenesis by favoring tumor cell proliferation.

### CXCR7 promotes melanoma proliferation through Src activation

**Fig. 3:**
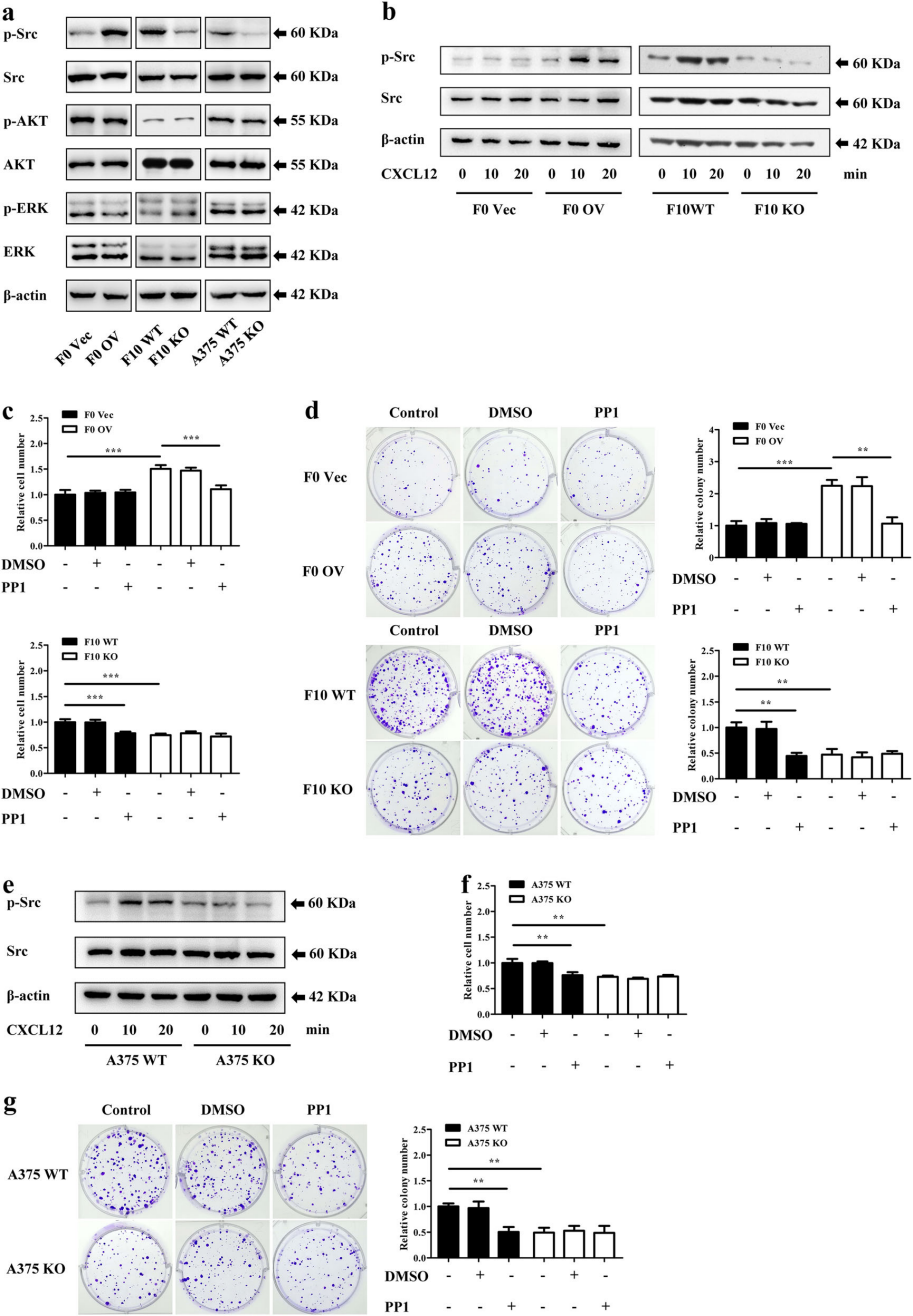
CXCR7 promotes melanoma proliferation through Src activation. **a** The effects of CXCR7 modifications on the Src, AKT, and ERK signalings. **b** CXCR7 expression modulated CXCL12-stimulated Src phosphorylation in the presence of AMD3100. Murine melanoma cell lines were cultured in serum-free medium overnight. After pretreated with AMD3100 (1 μg/ml) for 1 h, the cells were stimulated with recombinant murine CXCL12 (50 ng/ml). The phosphorylation levels of Src were determined by western blot. **c** PP1 suppressed CXCR7-induced cell proliferation. Melanoma cells were seeded into 96-well plates in the presence of DMSO or PP1 (10 μM). After 48 h, the numbers of cells were examined by CCK-8 assays. The proliferation rates were normalized to F0 Vec cells (top) or F10 WT cells (bottom) without treatment. **d** Representative images and quantitative results of the clonogenic growth of cells treated with DMSO or PP1 (10 μM). The results were normalized to F0 Vec cells (top) or F10 WT cells (bottom) without treatment. **e** Serum-starved A375 WT and KO cells were pretreated with AMD3100 (1 μg/ml) for 1 h, and stimulated with recombinant human CXCL12 (100 ng/ml). Cells were harvested for western blot analysis of the phosphorylated Src kinase. **f** PP1 recapitulated the disrupted cell proliferation induced by loss of CXCR7 in A375 cells. The proliferation rates were normalized to A375 WT cells without treatment. **g** Clonogenic growth of A375 WT and KO cells with the same treatments described in **d**. The results were normalized to A375 WT cells without treatment. Proliferation experiments and colony formation assays were independently repeated three times. Data are presented as mean ± SD. **p < 0.01, ***p < 0.001

CXCR7 has been shown to activate multiple downstream signalings, including ERK, AKT/mTOR, and Src kinase^28,38,39^. To better understand the signaling pathways by which CXCR7 regulates melanoma proliferation, we examined several selected pathways in the murine cells with manipulated CXCR7 expression. The results in Fig. 3a indicated that modulations of CXCR7 elicited an overt impact on the Src activity, whereas the AKT and ERK signalings remained unaffected. Similar results were obtained in human A375 cells (Fig. 3a). To further validate this CXCR7-mediated Src activation, the established cell lines were pretreated with the specific CXCR4 antagonist AMD3100 to exclude the signaling transduction through CXCR4, and followed by CXCL12 treatment. As shown in Figure S3a, CXCL12 stimulated the phosphorylation of Src even in the presence of AMD3100. The optimal concentration was determined as 50 ng/ml. Furthermore, overexpression of CXCR7 in B16-F0 cells promoted the CXCL12-stimulated Src phosphorylation, while elimination of this receptor abrogated Src activation in B16-F10 cells, confirming that Src kinase is a downstream target of CXCR7 (Fig. 3b). Moreover, the selective Src inhibitor PP1 abolished CXCR7-overexpression-promoted proliferation in B16-F0 cells (Fig. 3c). Similarly, CXCR7-depletion-induced proliferative defects were recapitulated by PP1 treatment (Fig. 3c). In addition, we examined clonogenic growth of the cells treated with PP1, and observed consistent results (Fig. 3d). While the apoptosis rates assessed by flow cytometry remained undisrupted after CXCR7 alterations or PP1 treatment (Figure S3b, S3c).

In keeping with the findings in murine cells, blockade of CXCR4 was unable to prevent CXCL12-triggered Src phosphorylation in A375 cells (Figure S3e), which was diminished by complete ablation of CXCR7 (Fig. 3e). Importantly, while PP1 suppressed A375 proliferation and clonogenic growth to a similar degree as loss of CXCR7, it did not alter the proliferative rate and colony formation ability of A375 KO cells (Fig. 3f, g). These phenomena occurred in the absence of changes in apoptosis rates (Figure S3d). Together, these evidences elucidate that CXCR7 activates Src kinase to promote melanoma cell proliferation.

### CXCR7 stimulates Src kinase phosphorylation through β-arrestin2

**Fig. 4:**
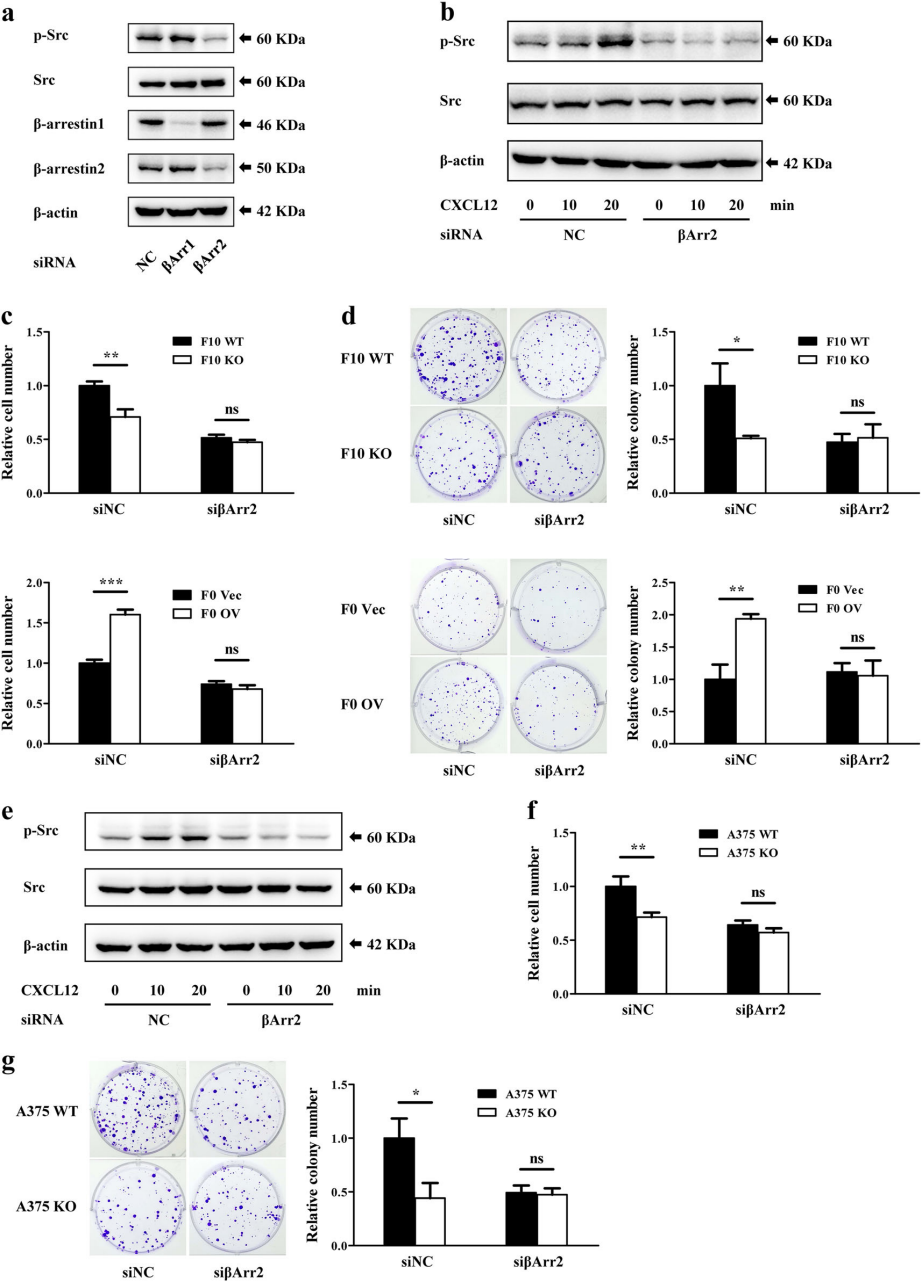
CXCR7 stimulates Src kinase phosphorylation through β-arrestin2. **a** The levels of Src phosphorylation in F10 WT cells transfected with siRNA targeting scramble control (NC), β-arrestin1 (βArr1), or β-arrestin2 (βArr2). **b**, **e** The effects of β-arrestin2 knockdown on CXCL12-induced Src phosphorylation in melanoma cells. F10 WT (**b**) and A375 WT (**e**) cells harboring scramble siRNA or β-arrestin2 siRNA were starved overnight. After pretreated with AMD3100 (1 μg/ml) for 1 h, the cells were exposed to recombinant murine (50 ng/ml) or human (100 ng/ml) CXCL12. The phosphorylation level of Src was detected by western blot. **c**, **f** The effects of β-arrestin2 downregulation on melanoma cell proliferation. Indicated cell lines were transfected with scramble siRNA or β-arrestin2 siRNA and seeded into 96-well plates. After 48 h, the cell numbers were determined by CCK-8 assays. The proliferation rates were normalized to F10 WT cells (**c**, top), F0 Vec cells (**c** bottom) or A375 WT cells (**f**) transfected with scramble siRNA. **d**, **g** The effects of β-arrestin2 downregulation on melanoma colony formation. Indicated cell lines were transfected with scramble siRNA or β-arrestin2 siRNA and seeded into 6-well plates. After 12 days, the colony numbers were counted. The quantitative results were normalized to F10 WT cells (**d**, top), F0 Vec cells (**d**, bottom) or A375 WT cells (**g**) transfected with scramble siRNA. Proliferation experiments and colony formation assays were independently repeated in triplicate. Data are presented as mean ± SD; *p < 0.05, **p < 0.01, ***p < 0.001; ns not significant

CXCR7 has been reported to activate downstream factors through β-arrestins in various cells^22^, yet their relationship in melanoma remains unclear. To explore the roles of β-arrestins in CXCR7-mediated Src activation in melanoma, we downregulated β-arrestin1 or β-arrestin2 by RNA interference (Figure S4a) and analyzed Src activities (Fig. 4a). The phosphorylation level of Src in B16-F10 cells was markedly attenuated by β-arrestin2 knockdown, but little affected by the inhibition of β-arrestin1. Moreover, siRNA targeting β-arrestin2 impaired the CXCL12-stimulated Src phosphorylation (Fig. 4b). To assess the contribution of β-arrestin2 to CXCR7-mediated melanoma proliferation, we suppressed β-arrestin2 expression in the constructed cell lines. With control siRNA transfection, F10 WT cells showed a higher proliferative rate than F10 KO cells. However, no significant difference was detected after the suppression of β-arrestin2 (Fig. 4c). Similarly, upregulation of CXCR7 endowed F0 OV cells with proliferation advantages, which was abrogated by β-arrestin2 knockdown (Fig. 4c). The importance of β-arrestin2 in CXCR7-mediated colonogenic growth was observed in colony formation assays (Fig. 4d). While no detectable differences were identified in apoptosis rates of the cell lines transfected with scramble or β-arrestin2 siRNA (Figure S4b, S4c).

In human A375 melanoma cells, administration of siRNA targeting β-arrestin2 resulted in a decreased Src activity (Figure S4d, S4e). Concurrently, upon CXCL12 stimulation, Src phosphorylation was substantially reduced by β-arrestin2 inhibition (Fig. 4e). In addition, A375 WT and KO cells displayed equivalent proliferative rates after downregulation of β-arrestin2 (Fig. 4f). Consistent results were obtained in clonogenic assays (Fig. 4g), while flow cytometry analysis indicated that β-arrestin2 silencing had no significant effects on cell apoptosis (Figure S4f). Collectively, these results demonstrate that β-arrestin2 is indispensable to CXCR7-induced Src kinase phosphorylation and cell proliferation in melanoma.

### CXCR7 contributes to melanoma angiogenesis and promotes VEGF secretion by upregulating HIF-1α expression

**Fig. 5:**
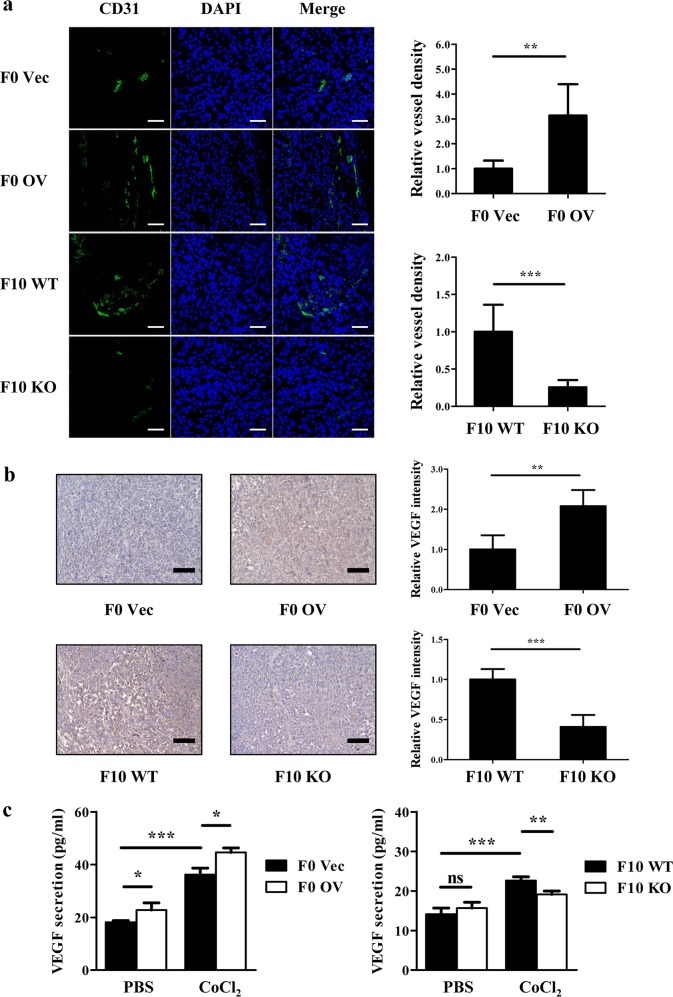

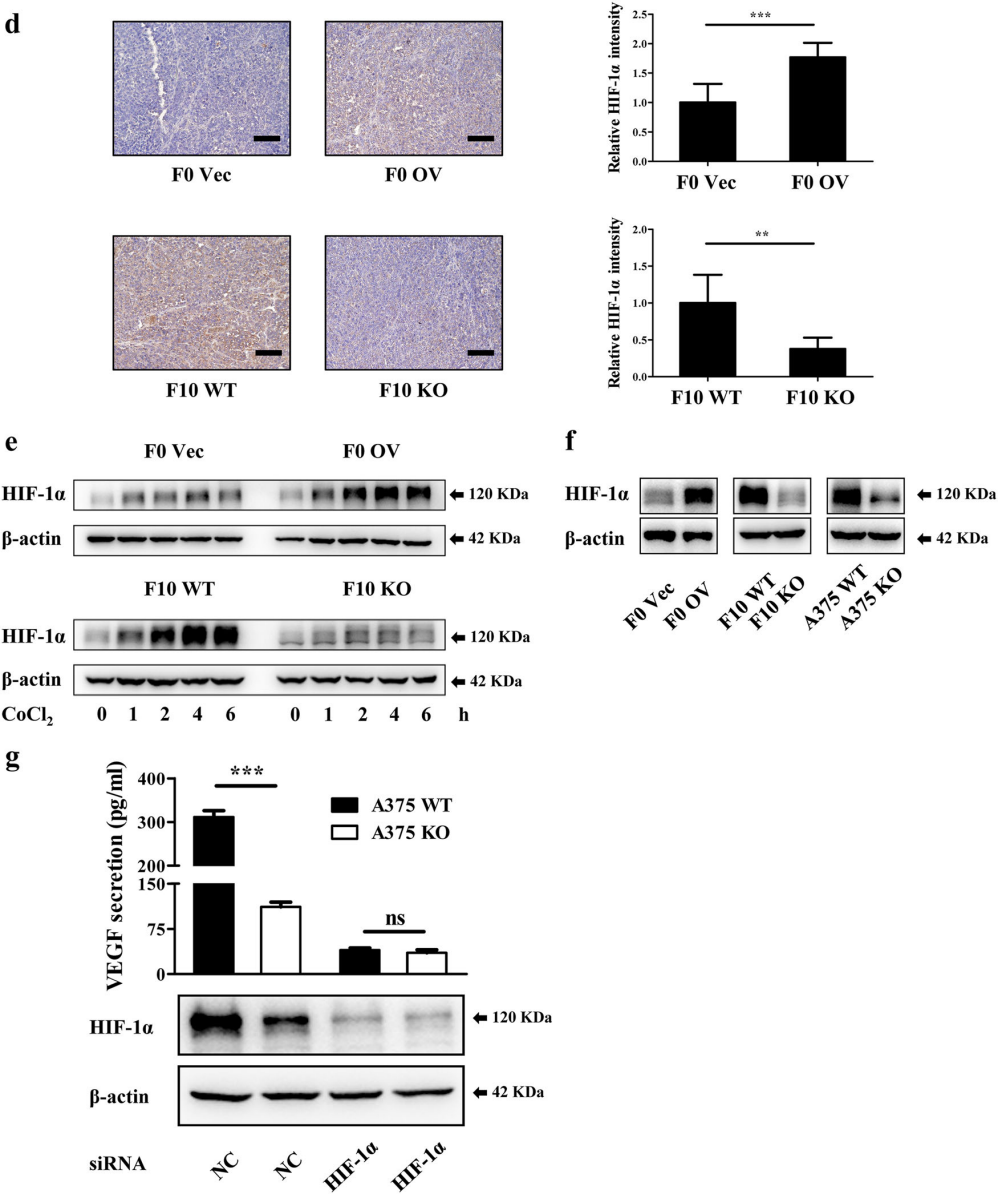
CXCR7 contributes to melanoma angiogenesis and promotes VEGF secretion by upregulating HIF-1α expression. **a** Immunofluorescent staining of CD31 (green) and DAPI (blue) in tumors derived from murine melanoma cells. The blood vessel densities were calculated by dividing CD31 area by DAPI area and were normalized to F0 Vec tumors (top) or F10 WT tumors (bottom). Left: representative images; scale bar = 50 μm. Right: relative quantitative results (*n* = 6–8 for each group). **b** Immunohistochemistry staining of VEGF in tumors derived from mice bearing murine melanoma cells. The staining intensities of VEGF were normalized to F0 Vec tumors (top) or F10 WT tumors (bottom). Left: representative images; scale bar = 100 μm. Right: relative quantitative results of staining intensities (*n* = 6–8 for each group). **c** The secretion of VEGF by murine melanoma cells with or without CoCl_2_ (200 μM) treatment. Cells were pretreated with CoCl_2_ for 6 h, and then incubated in serum-free medium containing CoCl_2_ overnight. The conditioned mediums were collected and the levels of VEGF were determined by ELISA. **d** Immunohistochemistry staining of HIF-1α in tumors derived from mice bearing murine melanoma cells. The staining intensities of HIF-1α were normalized to F0 Vec tumors (top) or F10 WT tumors (bottom). Left: representative images; scale bar = 100 μm. Right: relative quantitative results of staining intensities (*n* = 6–8 for each group). **e** HIF-1α expression in murine melanoma cells treated with CoCl_2_. **f** HIF-1α expression in melanoma cells under normoxic conditions. **g** A375 WT and KO cells transfected with siRNA targeting scramble control or HIF-1α were treated by CoCl_2_. The conditioned mediums and cell lysates were collected to detect VEGF secretion and HIF-1α expression. ELISA assays were independently conducted in triplicate. The quantification of immunofluorescent and immunohistochemistry staining were evaluated in 12 random fields for each tumor. Data are presented as mean ± SD, *p < 0.05, **p < 0.01, ***p < 0.001; ns not significant

Given the results shown in Fig. 2 that ablation of CXCR7 exerts more significant inhibitory effects on tumor growth in vivo than on cell proliferation in vitro, we wondered whether CXCR7 is implicated in melanoma angiogenesis. Immunofluorescent staining of CD31 on tumor sections revealed that CXCR7 overexpression triggered higher blood vessel densities, while depletion of CXCR7 led to a suppression of angiogenesis in F10 KO tumors (Fig. 5a). In addition, the expression level of the well-established pro-angiogenic factor, VEGF, was detected by immunohistochemistry staining and showed positive correlation with CXCR7 expression (Fig. 5b). To further evaluate the contribution of CXCR7 to VEGF secretion, we utilized CoCl_2_ as a mimic of hypoxia to treat cells and collected conditioned mediums to measure the release of VEGF. F0 OV cells secreted a higher level of VEGF than the control cells (Fig. 5c, left). Consistently, the supernatant level of VEGF in B16-F10 cells following CoCl_2_ incubation was significantly attenuated in the absence of CXCR7 (Fig. 5c, right). To verify these observations in human melanoma, the expressions of CD31 and VEGF were assessed in A375 WT and KO groups. As shown in Figure S5a–S5c, similar results were obtained both in vivo and in vitro.

As the master regulator during hypoxic responses, HIF-1α is required for the hypoxic regulation of VEGF secretion and tumor angiogenesis^40,41^. Increased expression of HIF-1α is correlated with VEGF upregulation and critical for melanoma progression^42,43^. Based on above findings, we hypothesized that CXCR7 should promote VEGF secretion via increasing HIF-1α expression. Indeed, immunohistochemistry staining showed a higher level of HIF-1α in tumors overexpressing CXCR7, while decreased expressions of HIF-1α were detected in CXCR7-depleted tumors (Fig. 5d, Figure S5b). In the context of CXCR7 overexpression, CoCl_2_ treatment in vitro enhanced HIF-1α accumulation in a time-dependent manner, while the level of HIF-1α was substantially compromised due to CXCR7 depletion (Fig. 5e, Figure S5d). Interestingly, overexpression or knockout of CXCR7 significantly promoted or attenuated HIF-1α expression even under normoxic conditions (Fig. 5f). To further validate that CXCR7 regulates VEGF secretion through HIF-1α, we downregulated HIF-1α in A375 WT and KO cells by siRNA transfection (Figure S5e). As shown in Fig. 5g, the secretion of VEGF was remarkably reduced in CXCR7-depleted cells. However, this difference was abrogated after the inhibition of HIF-1α. Thus, these results highlight the role for CXCR7 in melanoma angiogenesis and elucidate that CXCR7 facilitates VEGF secretion by elevating HIF-1α expression.

### CXCR7 accelerates HIF-1α translation by facilitating Src-mediated eIF4E phosphorylation

**Fig. 6:**
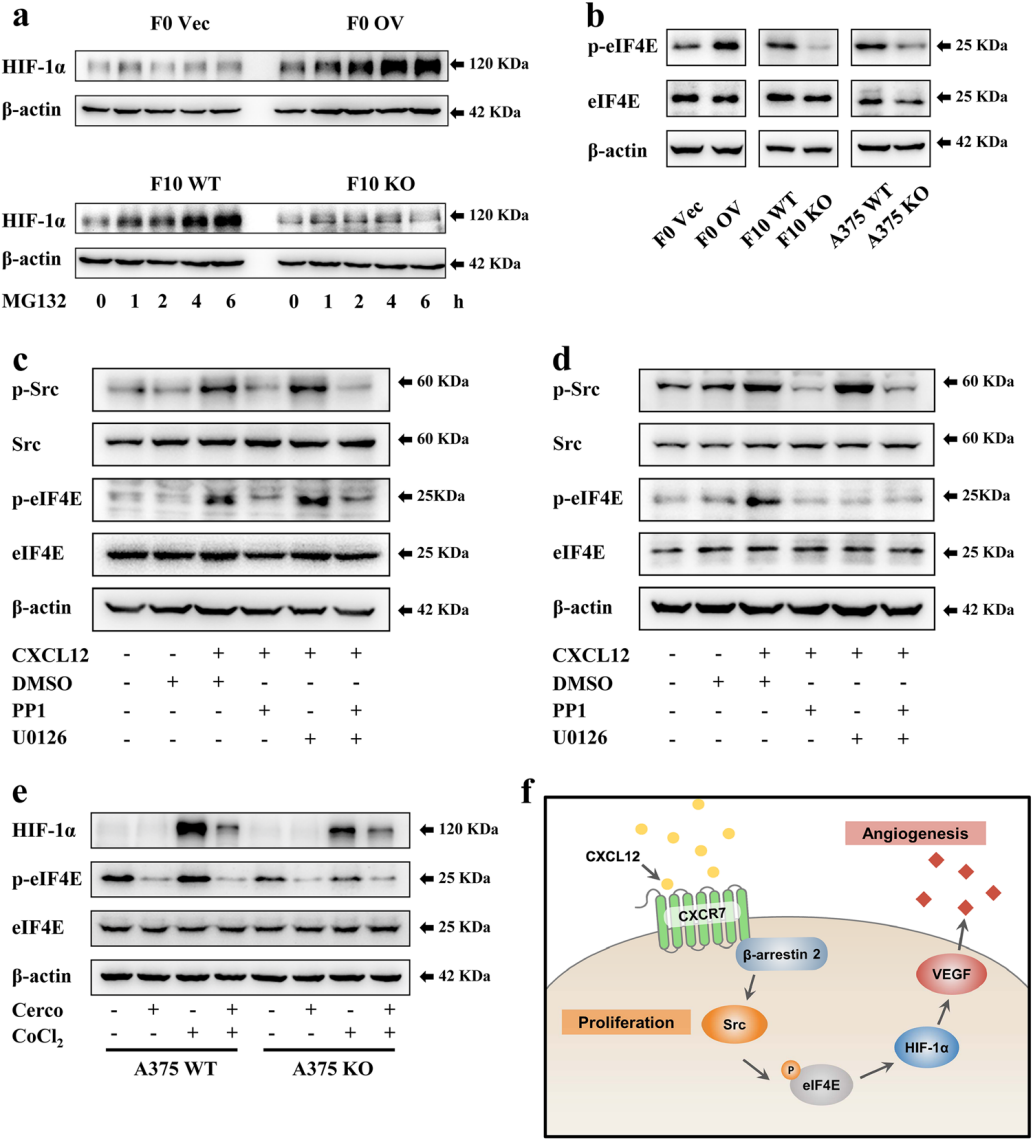
CXCR7 accelerates HIF-1α translation by facilitating Src-mediated eIF4E phosphorylation. **a** HIF-1α expression in murine melanoma cells treated with MG132 (10 μM). **b** The effects of CXCR7 modifications on eIF4E phosphorylation in melanoma cells. **c**, **d** CXCL12 stimulated eIF4E phosphorylation through the Src signaling in melanoma cells. F10 WT (**c**) and A375 WT (**d**) cells were starved overnight. After pretreated with AMD3100 (1 μg/ml) combined with PP1 (10 μM) or U0126 (10 μM) for 1 h, the cells were stimulated with recombinant murine (50 ng/ml) or human (100 ng/ml) CXCL12 as indicated. The cell lysates were collected and immunoblotted with the indicated antibodies. **e** A375 WT and KO cells were pretreated with cercosporamide (cerco, 20 μM) overnight and then incubated with CoCl_2_ (200 μM) for 4 h. The levels of HIF-1α and phosphorylated eIF4E were examined by western blot. **f** The proposed model for the functions of CXCR7 in melanoma tumorigenesis. CXCR7 is activated by CXCL12 to support melanoma cell proliferation through β-arrestin2-mediated Src phosphorylation. In addition, the activation of this CXCR7-Src axis stimulates eIF4E phosphorylation to accelerate the translation of HIF-1α, which enhances the secretion of VEGF to facilitate melanoma angiogenesis

Previous studies demonstrated that HIF-1α expression is orchestrated at multiple steps, including transcription, translation, and degradation^44^. Under normoxic conditions, newly synthesized HIF-1α is rapidly primed by the von Hippel-Lindau tumor suppressor protein (VHL) to proteasomal degradation^45^. Melanoma cells could stabilize HIF-1α by downregulation of VHL^46^. Thus, we detected HIF-1α transcription and VHL expression in all constructed cell lines and found minimal changes in each group (Figure S6a, S6b). Furthermore, to investigate whether CXCR7-induced HIF-1α accumulation was attributed to translational regulation, the proteasome inhibitor MG132 was administered to melanoma cells under normoxic conditions. The results showed that CXCR7 overexpression markedly potentiated HIF-1α accumulation, while the level of HIF-1α was robustly diminished in CXCR7-depleted cells (Fig. 6a, S6c). These findings suggest that CXCR7 promotes HIF-1α expression at the translational level.

Protein translation is a pivotal cellular process which is tightly regulated by various factors involved in multiple signaling pathways^47^. eIF4E is the rate-limiting regulator during mRNA translation, whose activity is modulated by phosphorylation at Ser-209 by MAP kinase-interacting kinase 1/2 (MNK1/2)^48^. Herein, we found that eIF4E phosphorylation was increased after CXCR7 overexpression, while opposite results were observed in CXCR7-depleted cells (Fig. 6b). To explore the contribution of Src kinase to CXCR7-induced eIF4E phosphorylation, PP1 was added to the medium when cells were stimulated with CXCL12. As shown in Fig. 6c, d, CXCL12-stimulated eIF4E phosphorylation was significantly impeded in murine and human cells incubated with PP1, demonstrating a reliance on Src activities. Intriguingly, the ERK inhibitor U0126 exhibited a similar inhibitory effect as PP1 did in A375 cells, yet only had an insignificant impact on B16-F10 cells. Furthermore, we administered an MNK1/2 inhibitor, cercosporamide^49^, to A375 WT and KO cells in the presence of CoCl_2_. As shown in Fig. 6e, CoCl_2_-induced HIF-1α accumulation in both groups were substantially suppressed to a comparable degree by inhibition of eIF4E phosphorylation. Therefore, our findings demonstrate that CXCR7 promotes HIF-1α translation in melanoma by facilitating Src-mediated eIF4E phosphorylation.

## Discussion

Many studies have explored the functions of CXCR7 in cancer^23,24^. However, the role of CXCR7 in melanoma remains elusive. Herein, we observed that CXCR7 was highly expressed in murine melanoma cells. Moreover, staining of CXCR7 in the patient tissue microarray showed that the level of CXCR7 was positively correlated with melanoma progression. Importantly, the overall survival analysis revealed that high CXCR7 expression is associated with worse overall survival in melanoma patients, indicating the potential prognostic value of CXCR7. Early studies reported that CXCR7 expression was mainly restricted to melanoma blood vessels^50,51^. In this study, our data demonstrated that direct modulations of CXCR7 expression in melanoma cells had significant effects on proliferation in vitro and tumor growth in vivo, indicating that CXCR7 potentiates melanoma tumorigenesis through regulating cell proliferation. Even though belonging to the G protein-coupled receptor family, CXCR7 is prevented from interacting with G-protein complexes due to alterations in the highly conserved DRY motif^19^. Some studies have reported that CXCR7 could function as a scavenger to maintain a concentration gradient of extracellular ligands^52–54^, whereas mounting evidences converge to a notion that CXCR7 is a fully signaling receptor to stimulate intracellular pathways through β-arrestins^22^. Here, we found that CXCL12-stimulated Src phosphorylation was regulated by CXCR7, which was abolished by downregulation of β-arrestin2. More importantly, CXCR7-induced melanoma cell proliferation was abrogated by the inhibition of Src kinase or β-arrestin2 expression. Thus, these findings reveal that CXCR7 is activated by CXCL12 to favor melanoma proliferation through β-arrestin2-mediated Src activation (Fig. 6f). In addition to melanoma cells, stromal fibroblasts and endothelial cells have also been documented as sources of CXCL12^55,56^. Thus CXCR7 may participate in the crosstalk between melanoma cells and stromal microenvironment. Recently, it has been reported that a ligand-independent activation of CXCR7 supports tumorigenesis through Src-mediated transactivation of EGFR^29,39,57^. However, our present data showed that EGFR expression was hardly detected in melanoma cells (Figure S3f), which is in consistent with previous studies^58,59^. Concordant with this, the EGFR inhibitor AG1478 displayed no impact on cell proliferation (Figure S3g–S3i), indicating that CXCR7 facilitates melanoma cell proliferation independent of EGFR.

CXCR7 signaling is known to be involved in tumor angiogenesis through facilitating VEGF secretion^26,28,38^. By using CD31 immunofluorescent staining, we observed that melanoma blood vessel densities were modulated by CXCR7 expression. Similarly, the results of immunohistochemistry staining and ELISA assays displayed that CXCR7 upregulated the secretion of VEGF in melanoma. In many types of cancer, HIF-1α is the vital regulator in tumor growth and angiogenesis, which is induced by hypoxic environment and oncogenic mutations to enhance VEGF production^40,41,45,60^. As shown here, the expressions of HIF-1α in melanoma tumor samples and in cells incubated with CoCl_2_ were modulated by CXCR7 expression. Intriguingly, early studies reported that melanoma cells could accumulate HIF-1α under normoxic conditions^43,61^, while this event is regulated in response to CXCR7 alterations, suggesting that CXCR7 is implicated in the abnormal expression of HIF-1α and hypoxic responses in melanoma. Furthermore, our results demonstrated that CXCR7-promoted VEGF secretion was abolished by inhibition of HIF-1α. Thus, CXCR7 supports melanoma angiogenesis through enhancing HIF-1α-mediated secretion of VEGF. Angiogenesis is a prominent hallmark of cancer^62^. Anti-angiogenesis therapies have been proved as efficacious treatments for melanoma patients^63,64^. Considering the high levels of CXCR7 in melanoma and many other types of cancer, it may serve as a promising target for the development of new anti-angiogenesis therapies.

The accumulation of HIF-1α has been documented in a variety of malignant tumors, including melanoma^42,45^. HIF-1α expression is elaborately modulated at multiple steps^45^. Our discoveries that manipulations of CXCR7 have minimal impact on HIF-1α mRNA level and VHL expression, whereas significantly modulate HIF-1α accumulation in the presence of the proteasome inhibitor MG132, suggest that CXCR7 promotes HIF-1α translation in melanoma cells. Protein translation is a pivotal biological process that is elevated by various signaling pathways to support uncontrolled cell proliferation in cancer^47^. The PI3K/AKT/mTOR axis has been considered as the central pathway governing translation through two major effectors, S6K and 4E-BP1^65^. However, in line with the undisturbed AKT signaling, the phosphorylations of S6K and 4E-BP1 remained unaffected in the context of CXCR7 modifications (Figure S6b). Of the four steps during protein translation, initiation is known as the rate-limiting process, in which eIF4E functions as the core regulator^66^. Emerging evidences have shown that the phosphorylation of eIF4E at Ser-209 has crucial roles in regulating its function and activity^48,67^. Here, our results showed that the phosphorylation level of eIF4E was associated with CXCR7 expression in melanoma cells. Moreover, CXCL12-stimulated eIF4E phosphorylation was substantially inhibited by PP1, demonstrating that CXCR7 promotes eIF4E phosphorylation through Src kinase. Currently, it is believed that eIF4E is exclusively phosphorylated by MAPK downstream kinases MNK1/2^68–71^. Interestingly, although the ERK inhibitor U0126 totally blocked eIF4E phosphorylation in human A375 cells, it had no influence on murine B16-F10 cells. Similarly, it has been reported that U0126 cannot inhibit eIF4E phosphorylation in T-cell lymphoma and hepatocellular carcinoma^72,73^. Taken together, these various lines of evidences point to a notion that the mechanisms whereby eIF4E is phosphorylated in cancer cells are complicated and require further investigations.

Defective phosphorylation of eIF4E has been shown to exhibit no significant effect on global protein translation^71^. In contradistinction, the syntheses of a subset of pro-tumor factors are sensitive to eIF4E phosphorylation^74,75^. HIF-1α expression was reported to be translationally regulated by the MNKs/eIF4E pathway^76,77^. Here we found that the MNK1/2 inhibitor cercosporamide diminished CoCl_2_-induced HIF-1α accumulation in melanoma cells, indicating that eIF4E phosphorylation is required for CXCR7-facilitated translation of HIF-1α. While eIF4E phosphorylation has been shown to be dispensable for normal development and fertility in genetically engineered mouse models, it is critical for cancer development and progression, making it an attractive target for cancer treatment^71,74,78,79^. In melanoma patients, eIF4E phosphorylation is known to be elevated and associated with poorer prognosis^80^. Moreover, cercosporamide inhibits B16 cell proliferation and the outgrowth of lung metastases^49^. Therefore, by demonstrating that CXCR7 is capable of accelerating HIF-1α translation by promoting Src-mediated eIF4E phosphorylation (Fig. 6f), our findings raise the possibility of interfering with this axis as clinical therapeutics for melanoma patients.

## Supplementary information

Figure S1

Figure S2–1

Figure S2–2

Figure S3

Figure S4

Figure S5

Figure S6

Figure S7-S17

Supplementary table

Informed Patient Consent

supplemental figure legends
