# Supplementary figures and images for: CXCR7 promotes melanoma tumorigenesis via Src kinase signaling
Source: Cell Death Dis. 2019 Feb 25;10(3):191. doi: 10.1038/s41419-019-1442-3 (PMC6389959; doi:10.1038/s41419-019-1442-3)

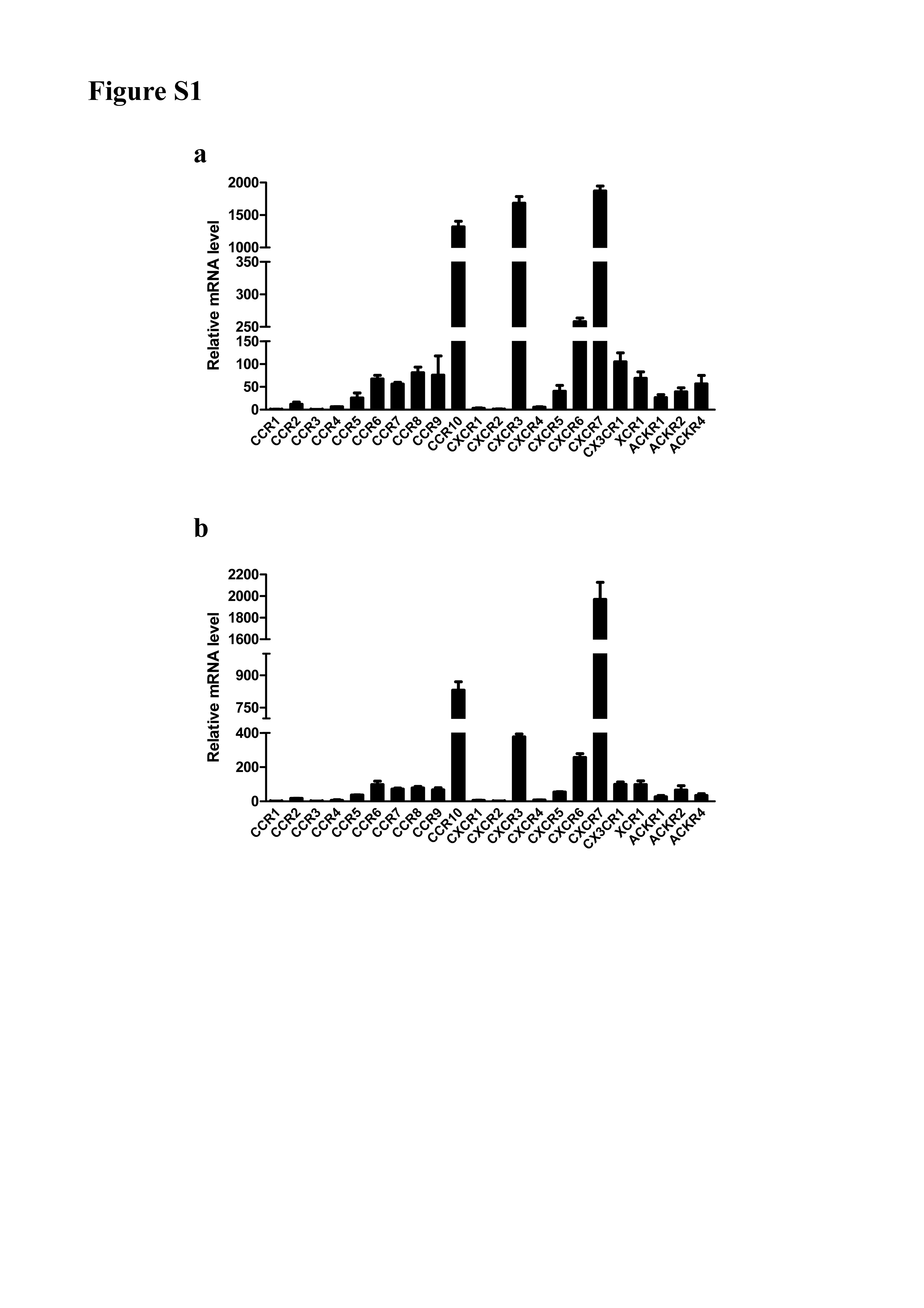

Supplement: Supplementary file 1 — Figure S1 [file 41419_2019_1442_MOESM1_ESM.tif]

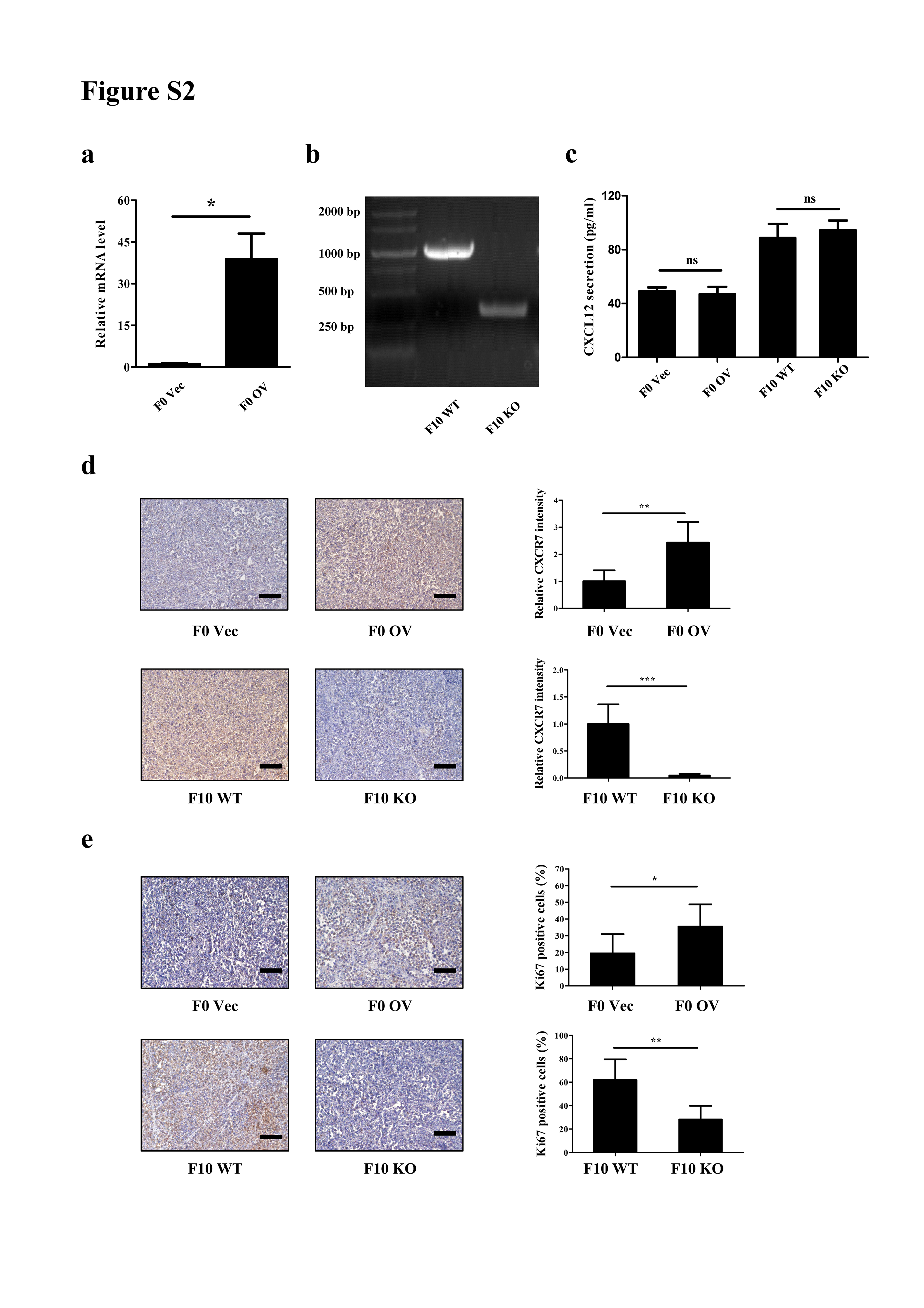

Supplement: Supplementary file 2 — Figure S2–1 [file 41419_2019_1442_MOESM2_ESM.tif]

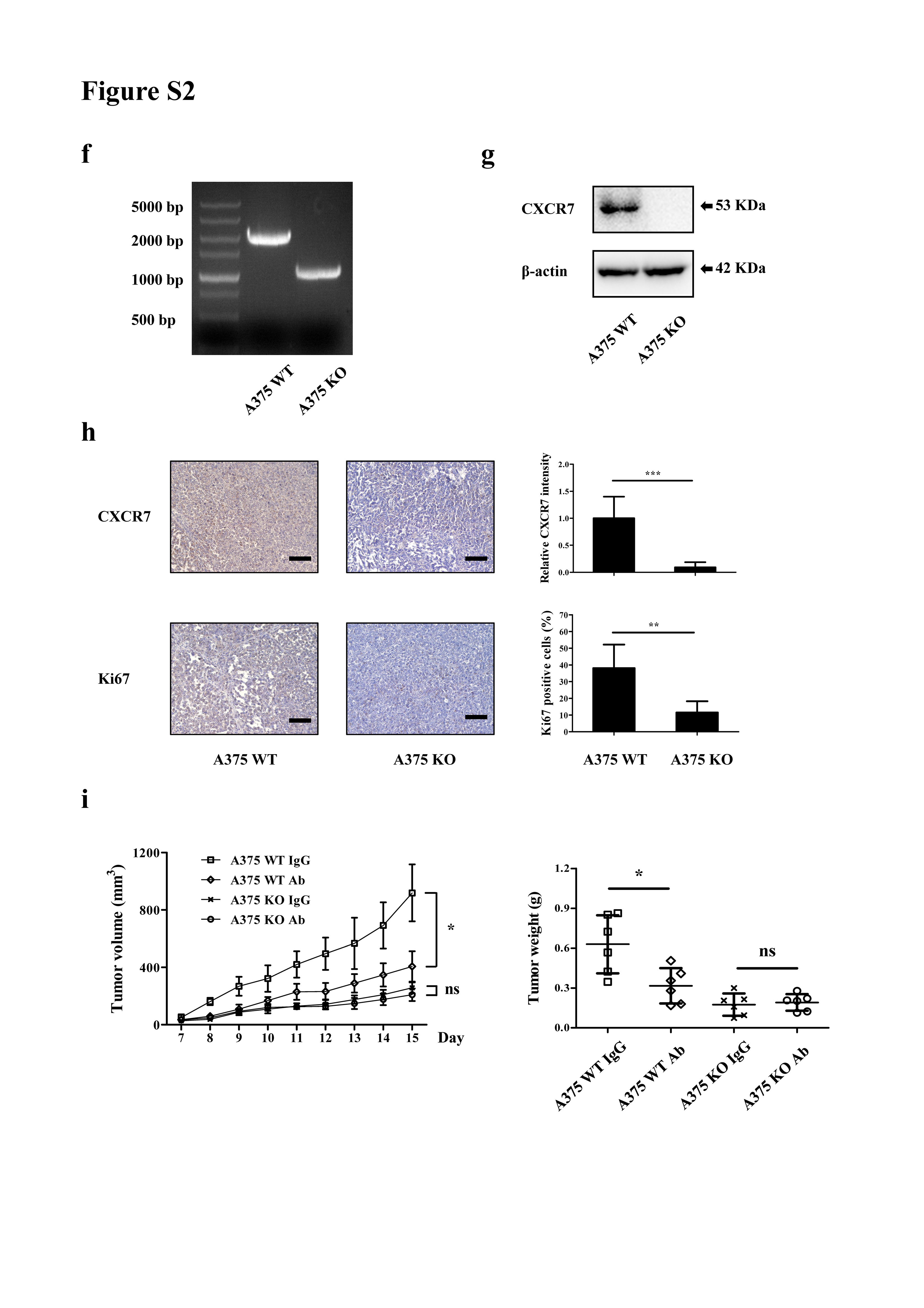

Supplement: Supplementary file 3 — Figure S2–2 [file 41419_2019_1442_MOESM3_ESM.tif]

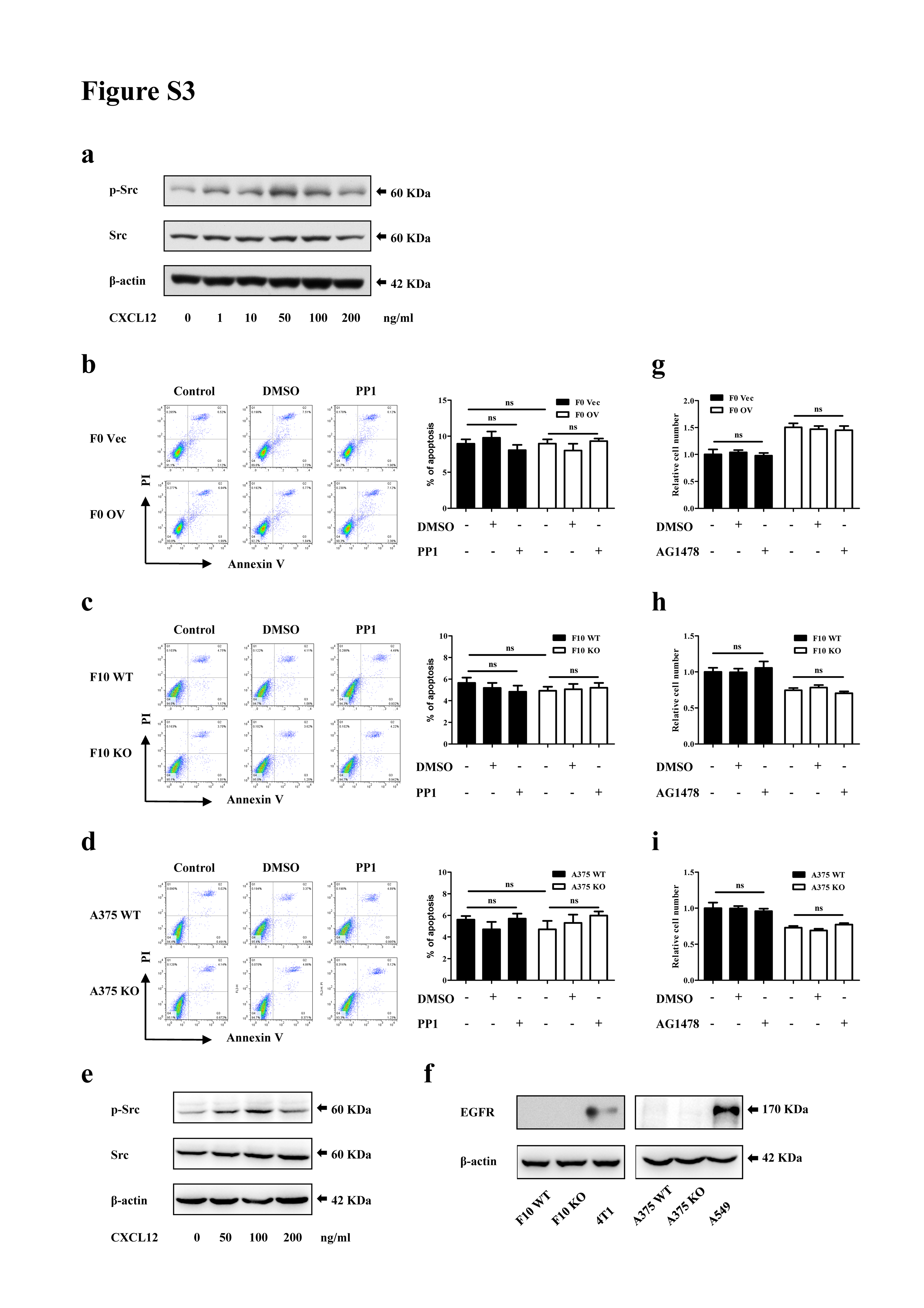

Supplement: Supplementary file 4 — Figure S3 [file 41419_2019_1442_MOESM4_ESM.tif]

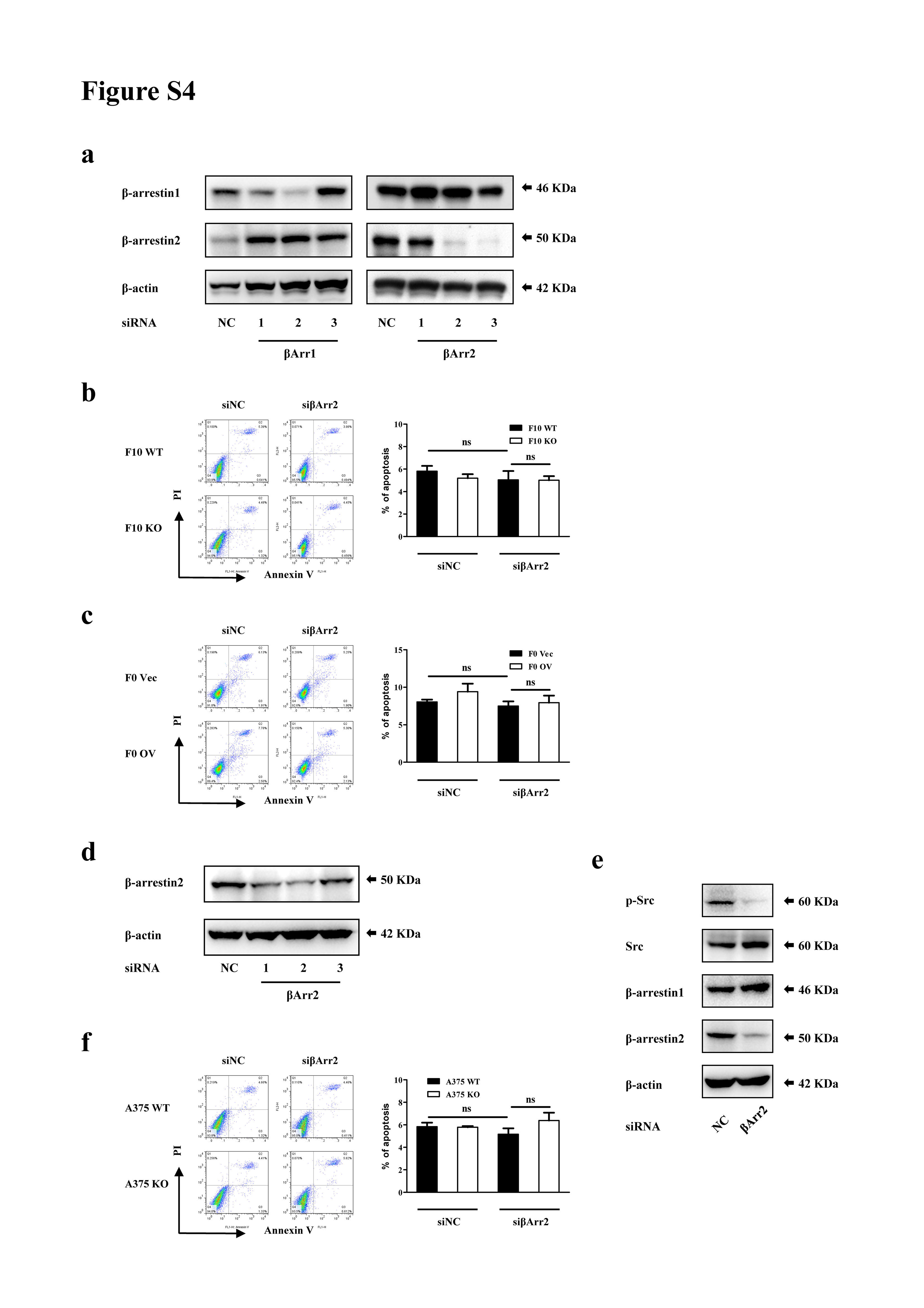

Supplement: Supplementary file 5 — Figure S4 [file 41419_2019_1442_MOESM5_ESM.tif]

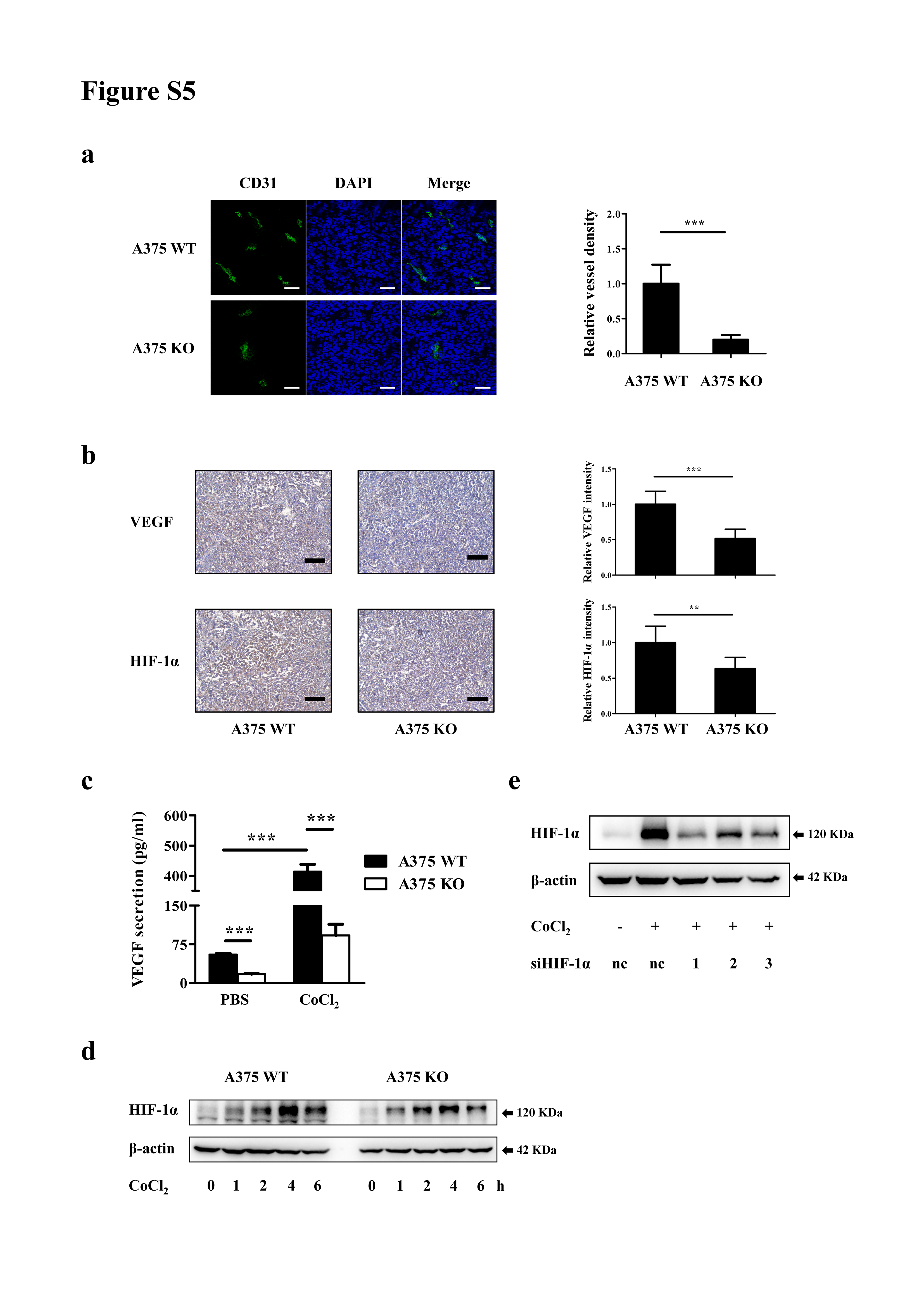

Supplement: Supplementary file 6 — Figure S5 [file 41419_2019_1442_MOESM6_ESM.tif]

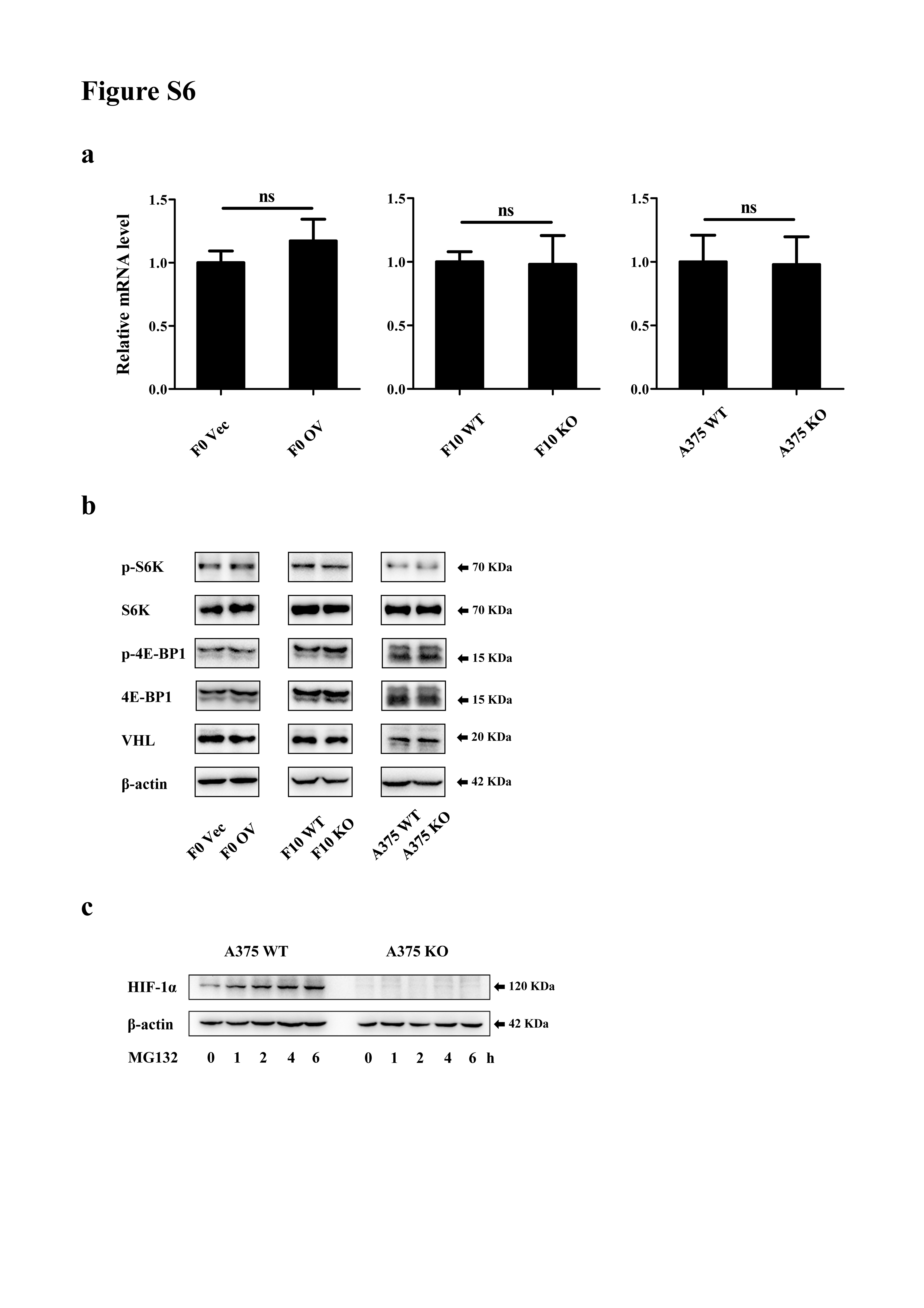

Supplement: Supplementary file 7 — Figure S6 [file 41419_2019_1442_MOESM7_ESM.tif]
