# Supplementary material for: CXCR7 promotes melanoma tumorigenesis via Src kinase signaling
Source: Cell Death Dis. 2019 Feb 25;10(3):191. doi: 10.1038/s41419-019-1442-3 (PMC6389959; doi:10.1038/s41419-019-1442-3)
Supplement: Supplementary file 8 — Figure S7-S17 [file 41419_2019_1442_MOESM8_ESM.pdf]

Figure S7

Figure 1c

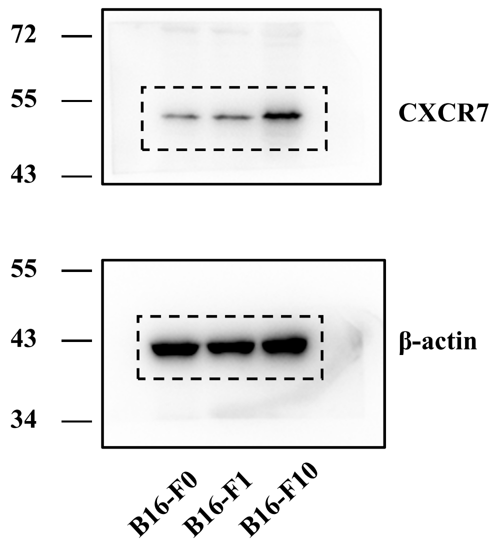

Figure 2a

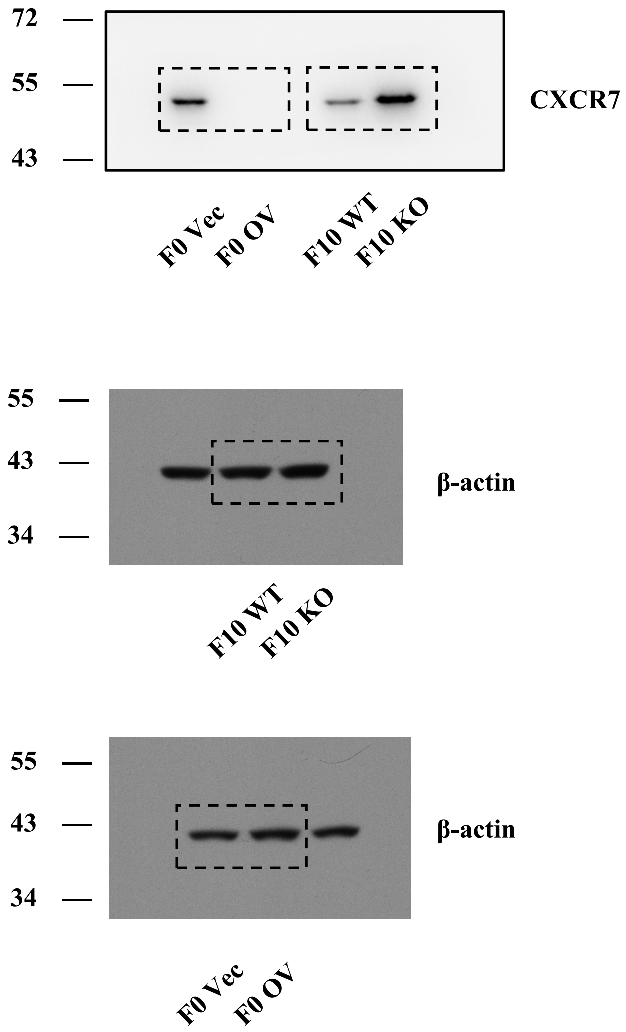

Figure S2g

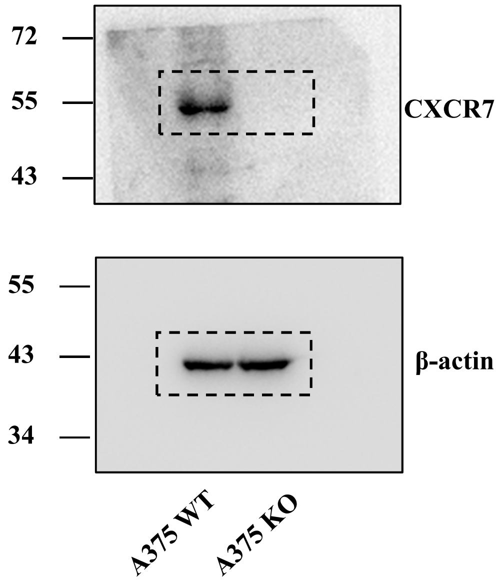

Uncropped Western blotting results in Figure 1c, Figure 2a and Figure S2g. Membranes were often cut to enable blotting with multiple primary antibodies.

Figure S8

Figure 3a

F0 Vec	F0 OV	F10 WT	F10 KO	A375 WT	A375 KO
1	2	3	4	5	6

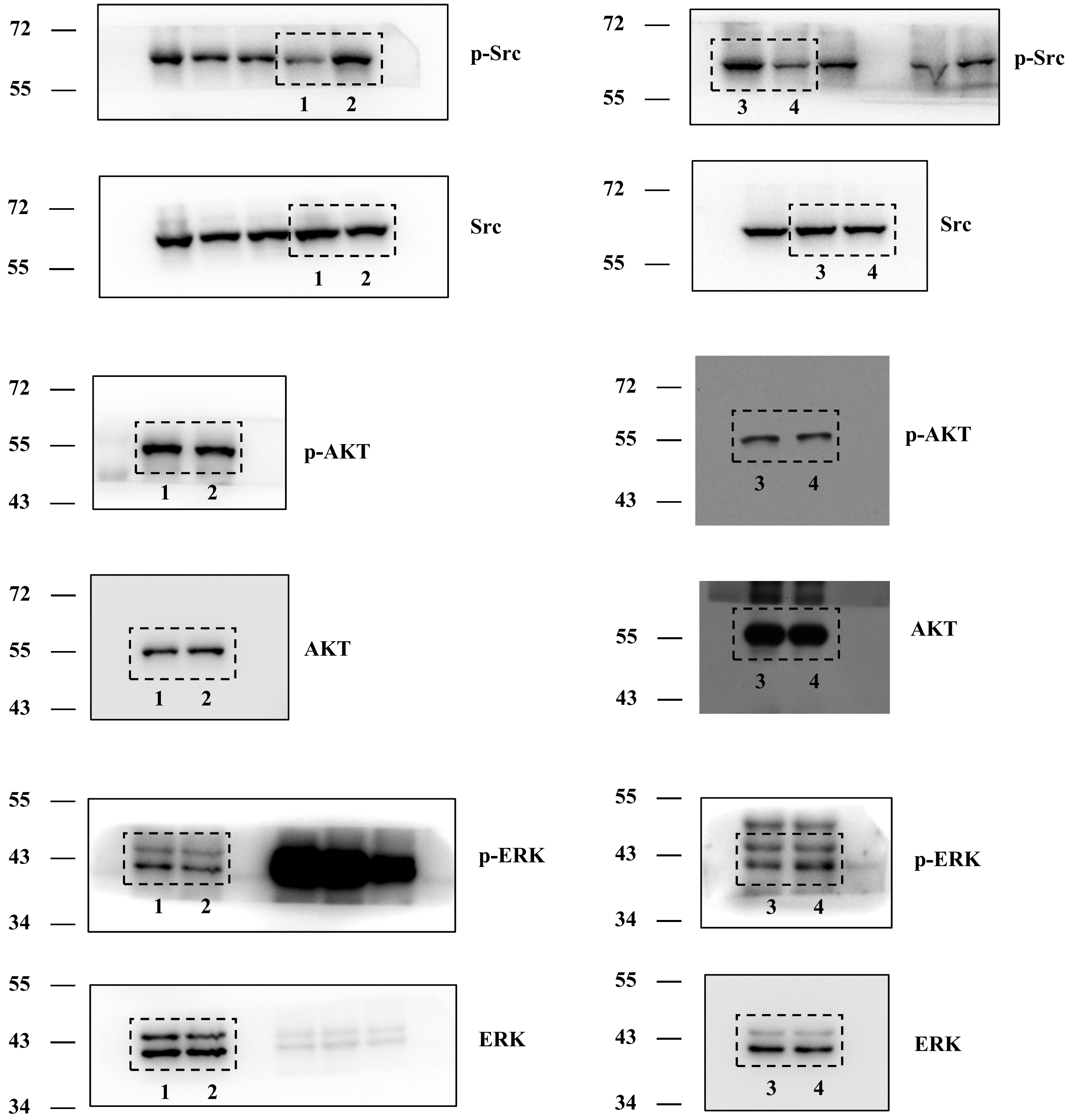

Uncropped Western blotting results in Figure 3a.
Membranes were often cut to enable blotting with multiple primary antibodies.

Figure S8

Figure 3a

F0 Vec	F0 OV	F10 WT	F10 KO	A375 WT	A375 KO
1	2	3	4	5	6

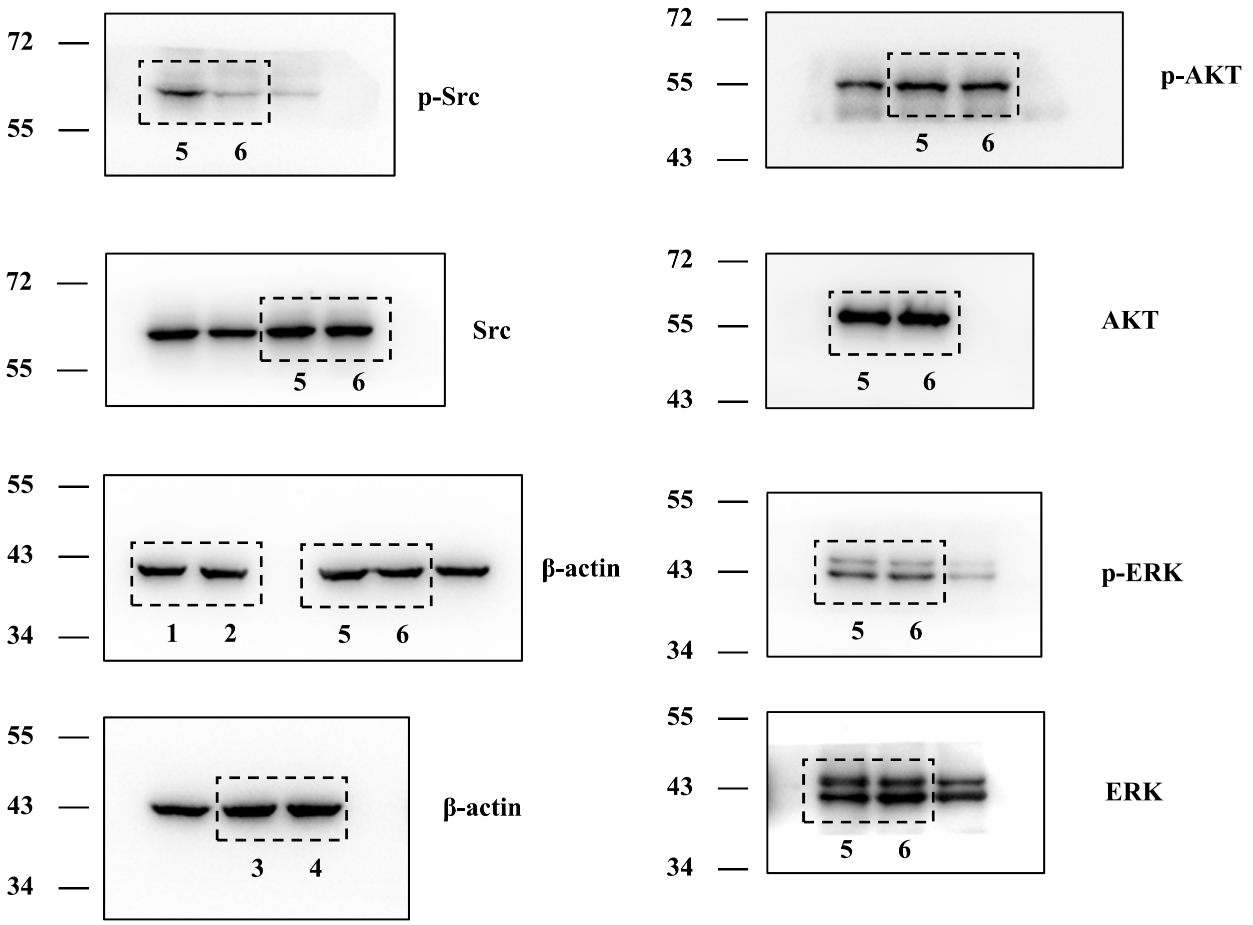

Uncropped Western blotting results in Figure 3a.
Membranes were often cut to enable blotting with multiple primary antibodies.

Figure S9

Figure 3b

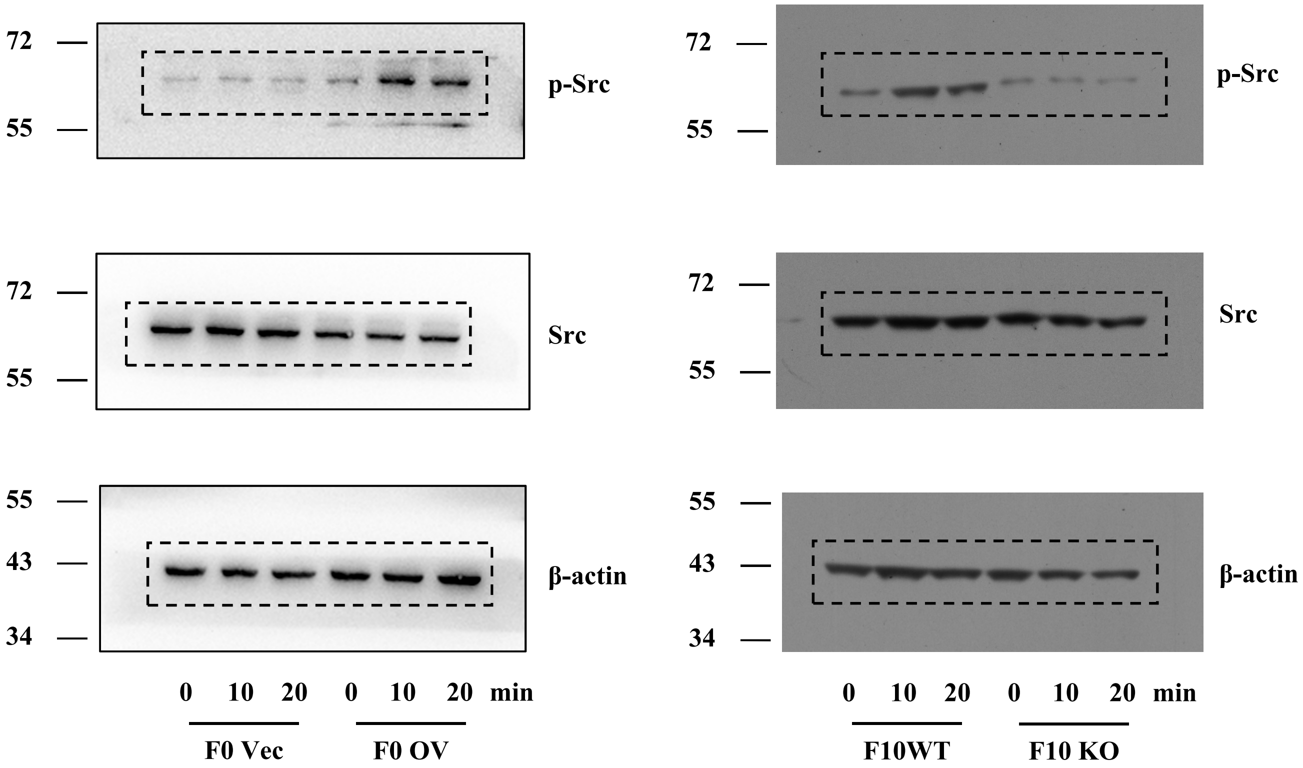

Figure 3e

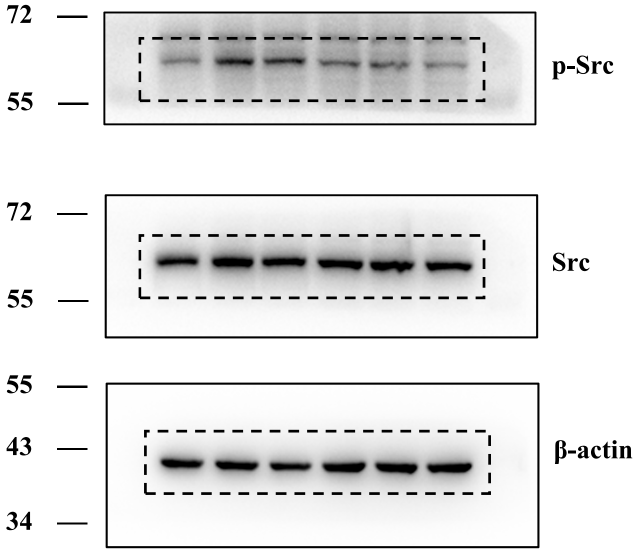

Uncropped Western blotting results in Figure 3b and Figure 3e.
Membranes were often cut to enable blotting with multiple primary antibodies.

Figure S10

Figure S3a

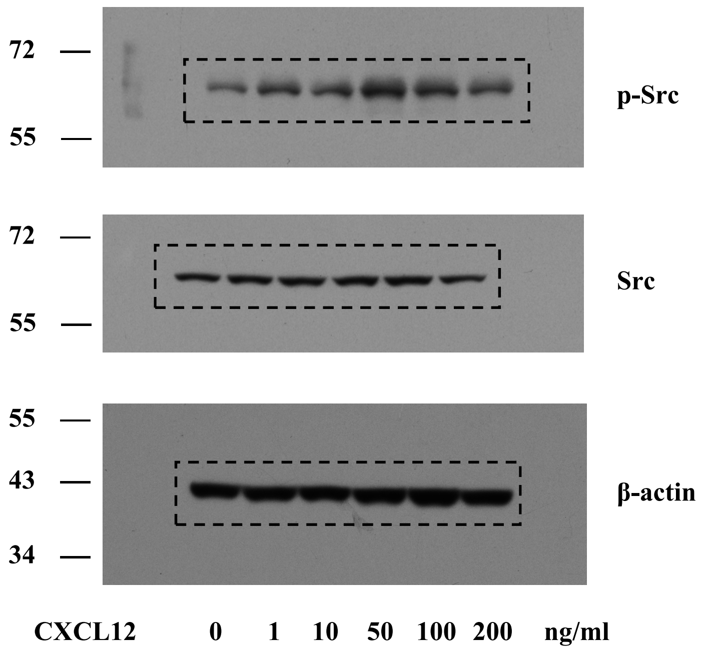

Figure S3e

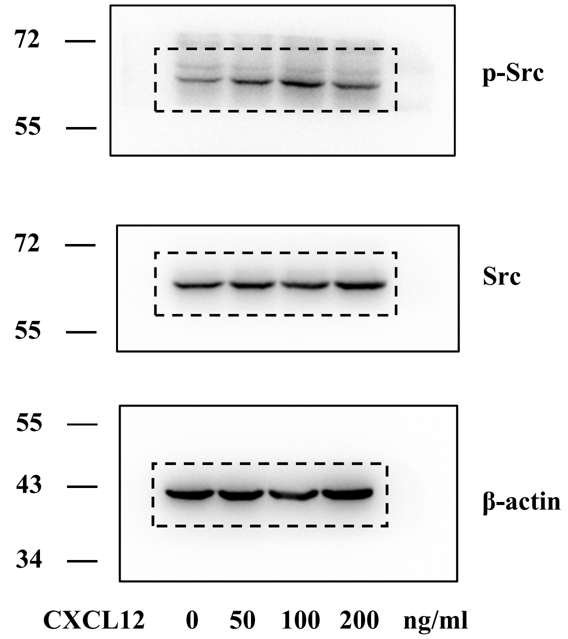

Figure S3f

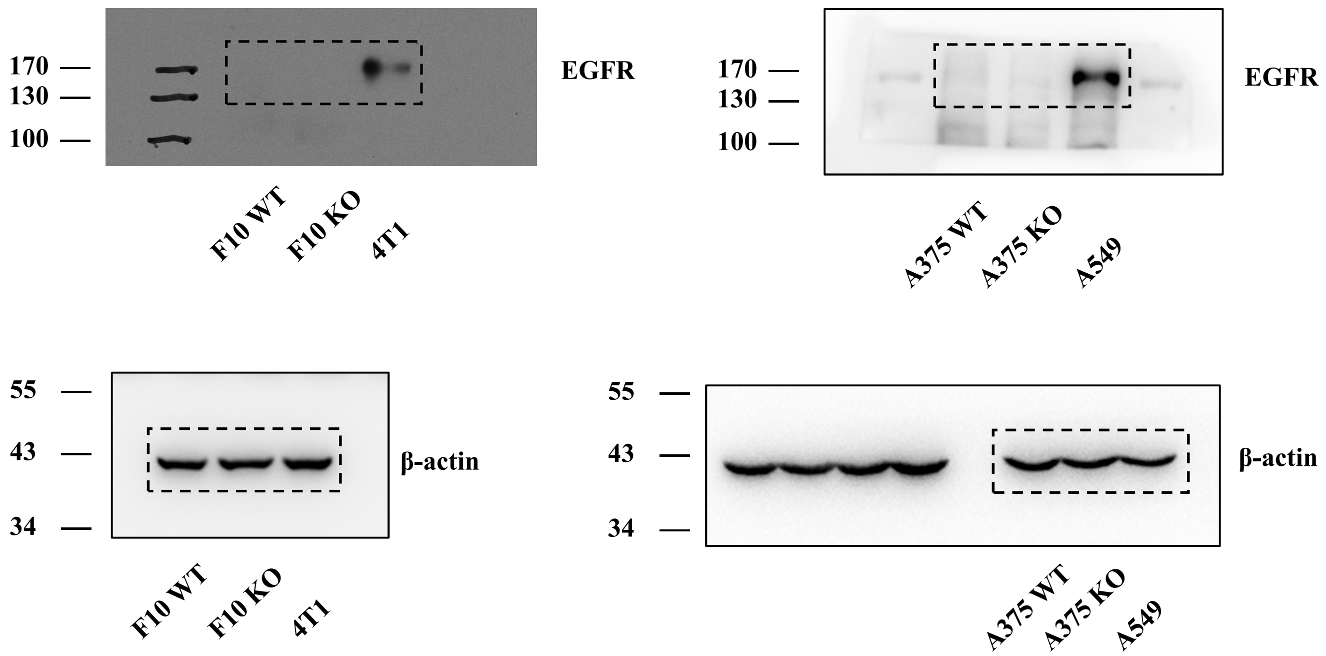

Uncropped Western blotting results in Figure S3a, Figure S3e and Figure S3f.
Membranes were often cut to enable blotting with multiple primary antibodies.

Figure S11

Figure 4a

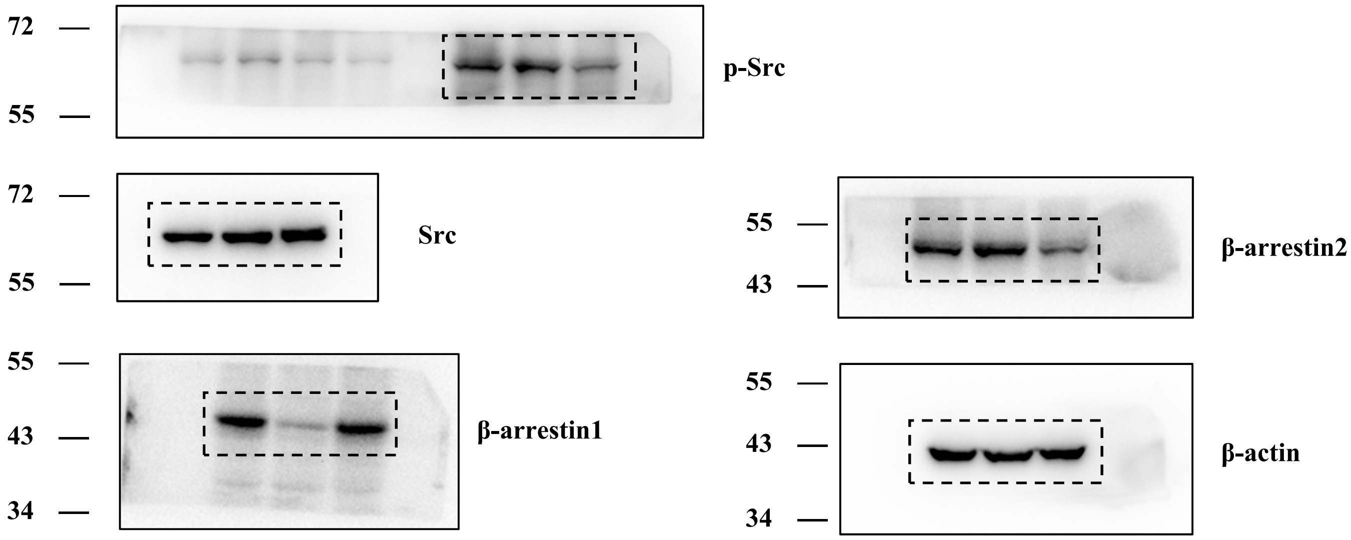

Figure 4b

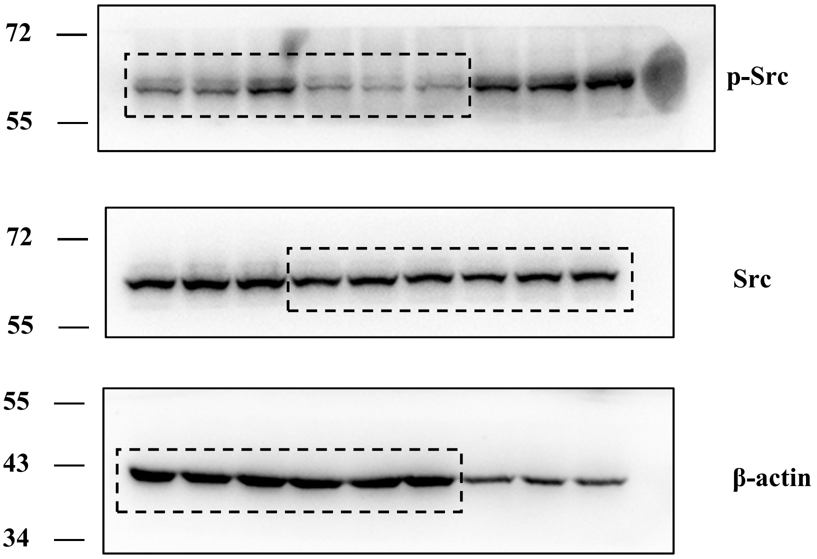

Figure 4e

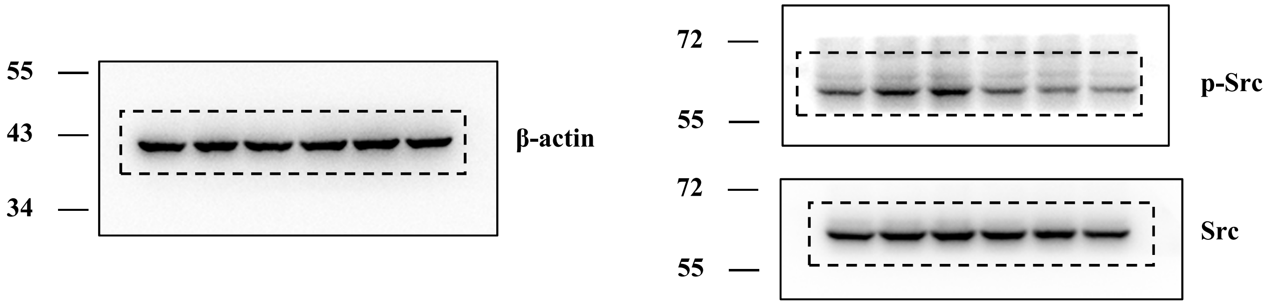

Uncropped Western blotting results in Figure 4a, Figure 4b and Figure 4e.
Membranes were often cut to enable blotting with multiple primary antibodies.

Figure S12

Figure S4a

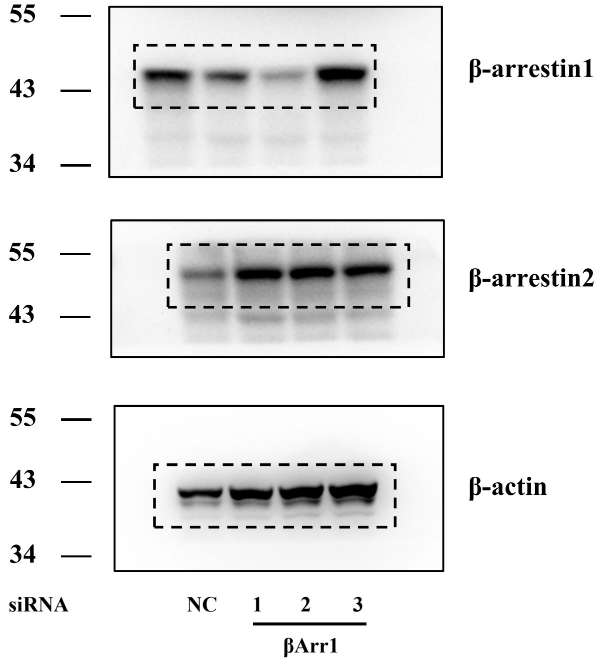

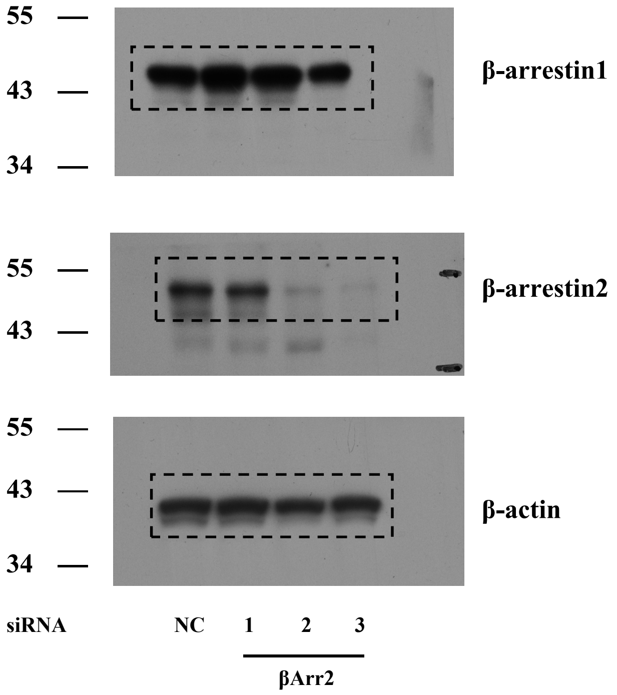

Figure S4d

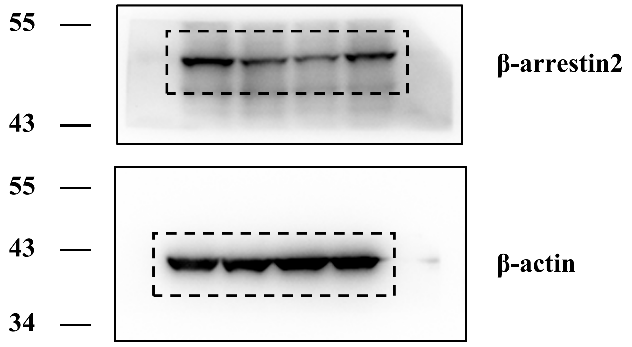

Figure S4e

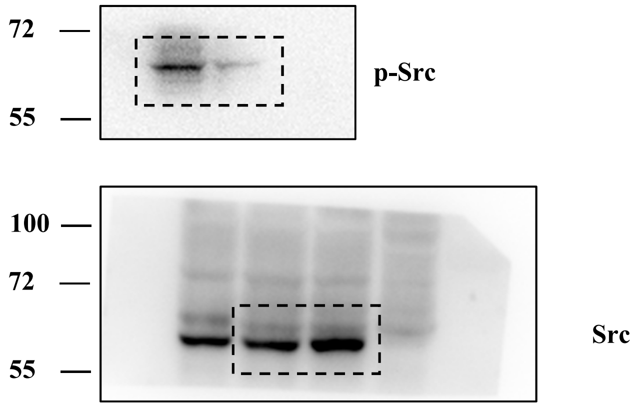

Figure S4e

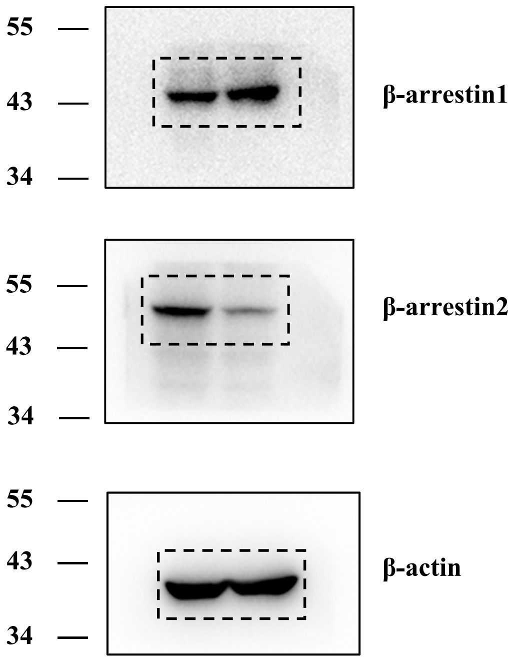

Uncropped Western blotting results in Figure S4a, Figure S4d and Figure S4e.
Membranes were often cut to enable blotting with multiple primary antibodies.

Figure S13

Figure 5e

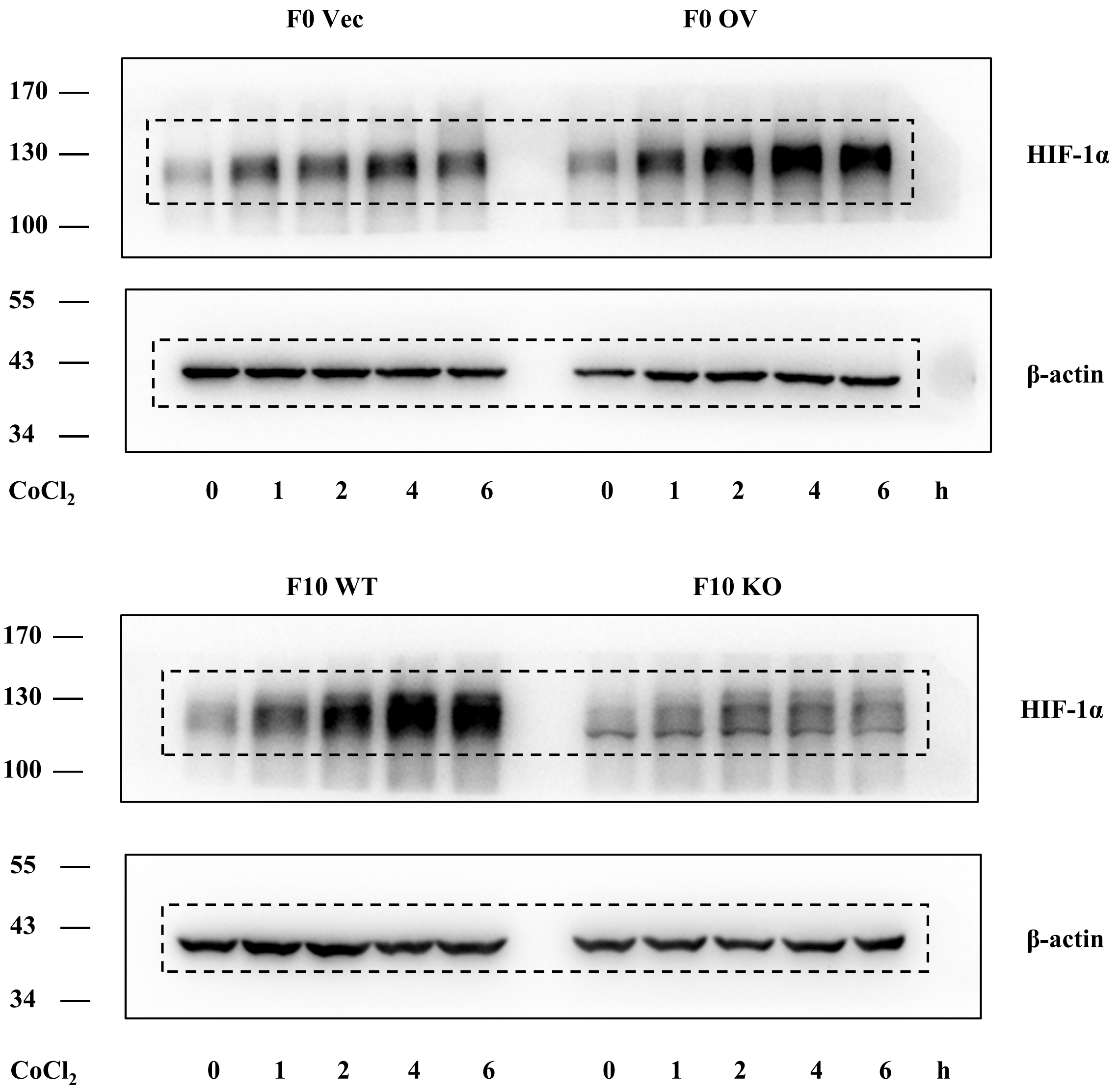

Figure 5g

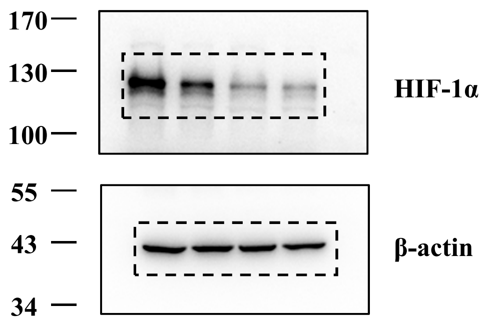

Figure S5e

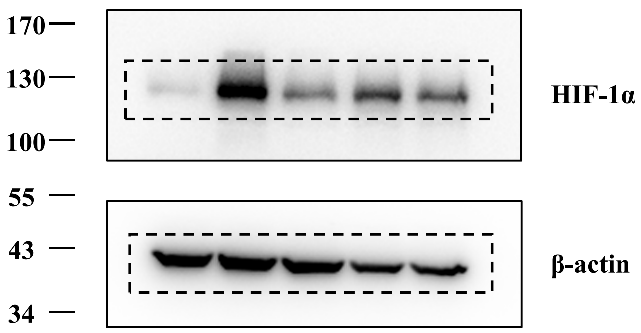

Uncropped Western blotting results in Figure 5e, Figure 5g and Figure S5e. Membranes were often cut to enable blotting with multiple primary antibodies.

Figure S14

Figure 5f

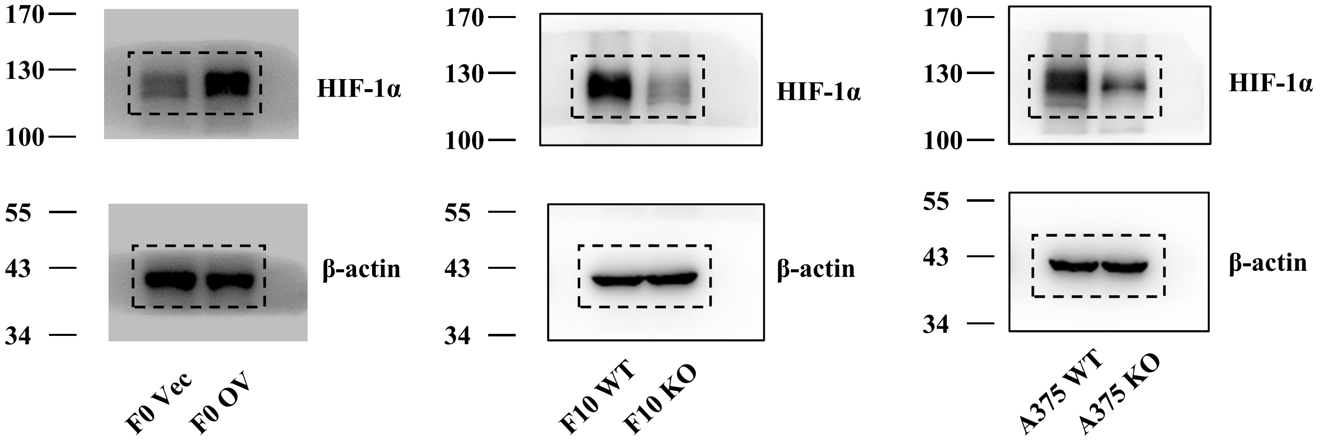

Figure S5d

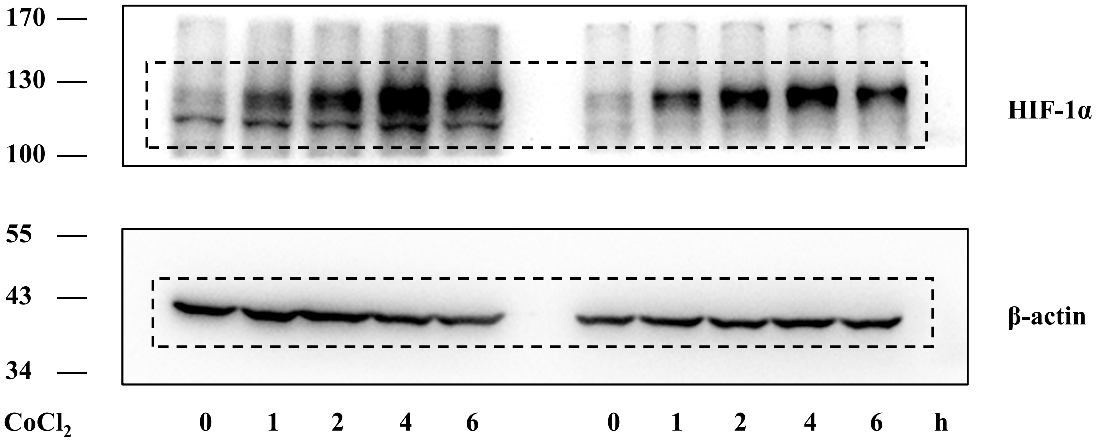

Figure 6c

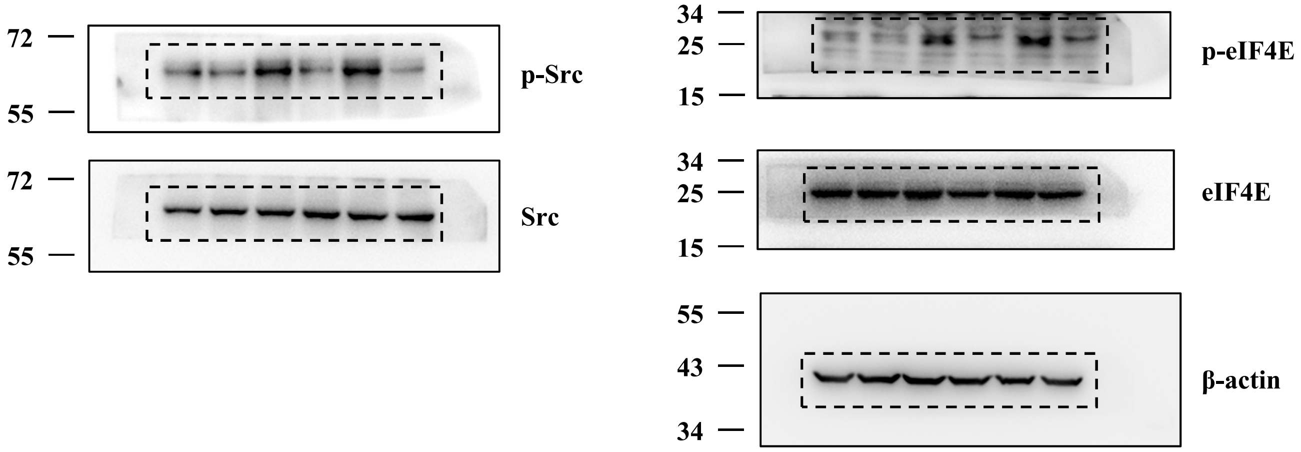

Uncropped Western blotting results in Figure 5f, Figure S5d and Figure 6c.
Membranes were often cut to enable blotting with multiple primary antibodies.

Figure S15

Figure 6a

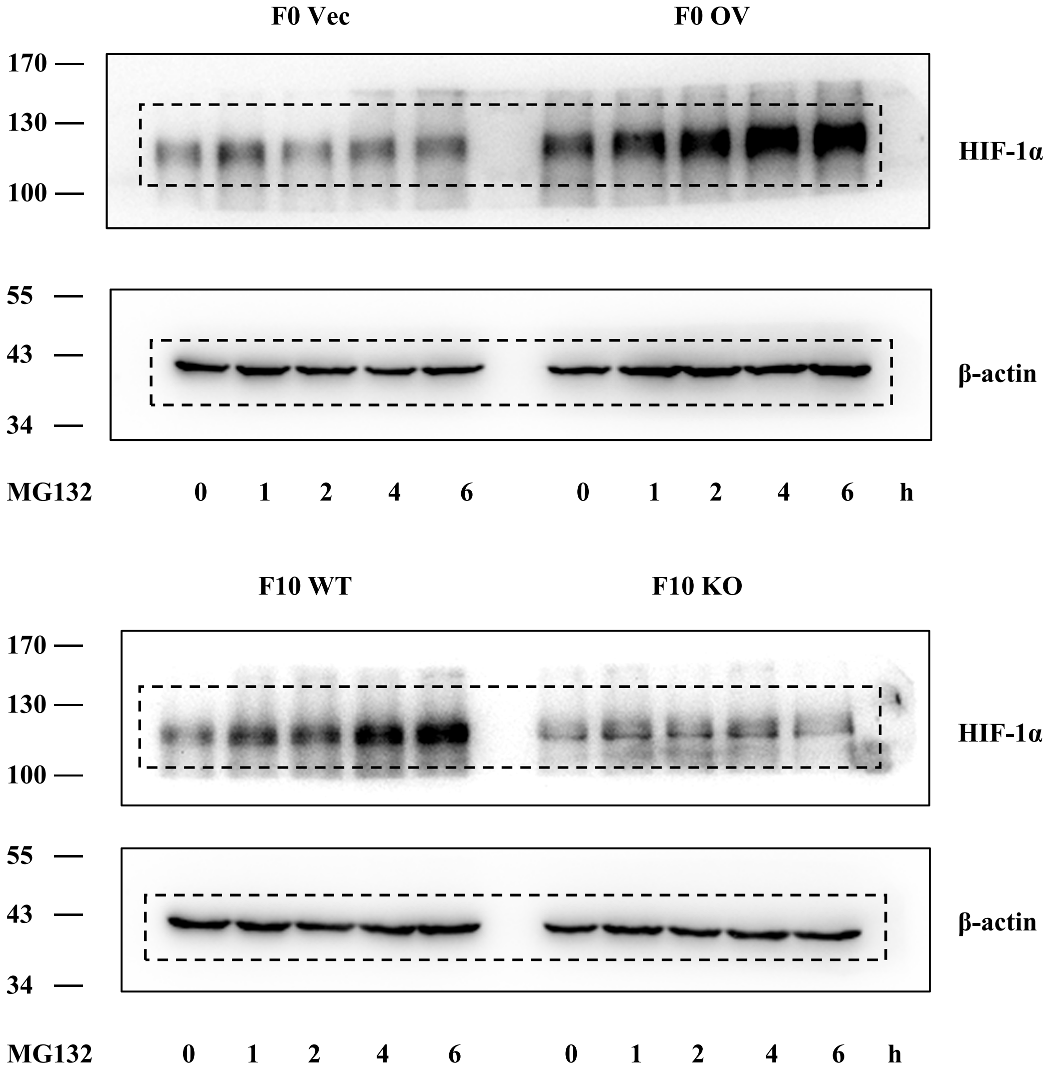

Figure 6d

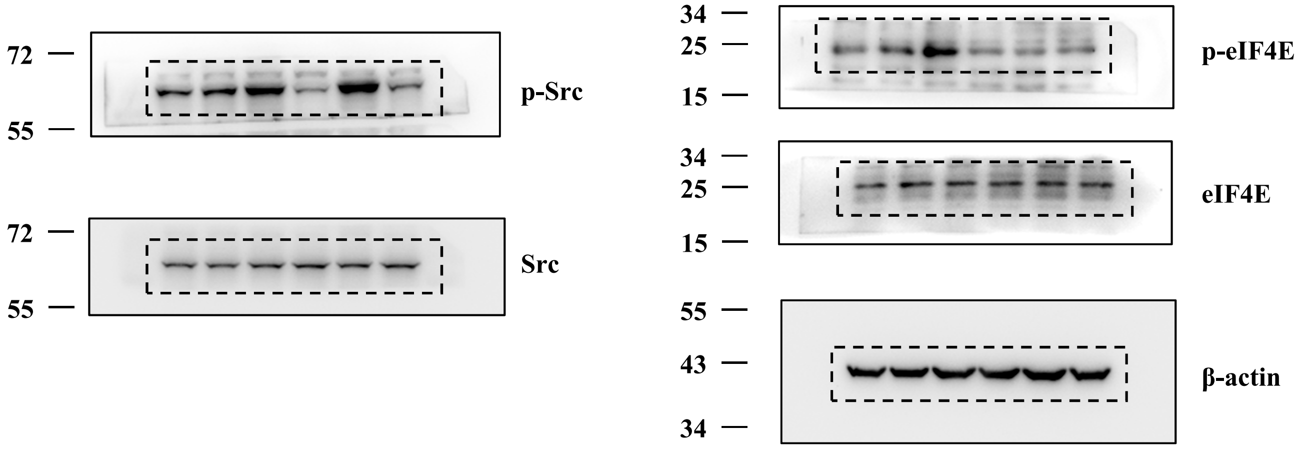

Uncropped Western blotting results in Figure 6a and Figure 6d.
Membranes were often cut to enable blotting with multiple primary antibodies.

Figure S16

Figure 6b

F0 Vec	F0 OV	F10 WT	F10 KO	A375 WT	A375 KO
1	2	3	4	5	6

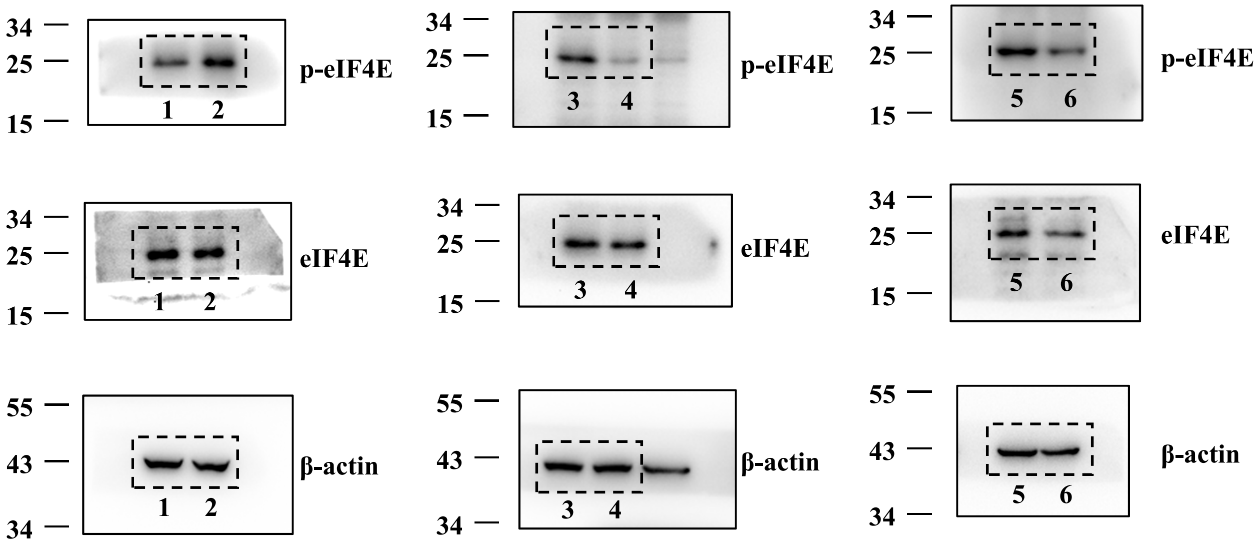

Figure S6c

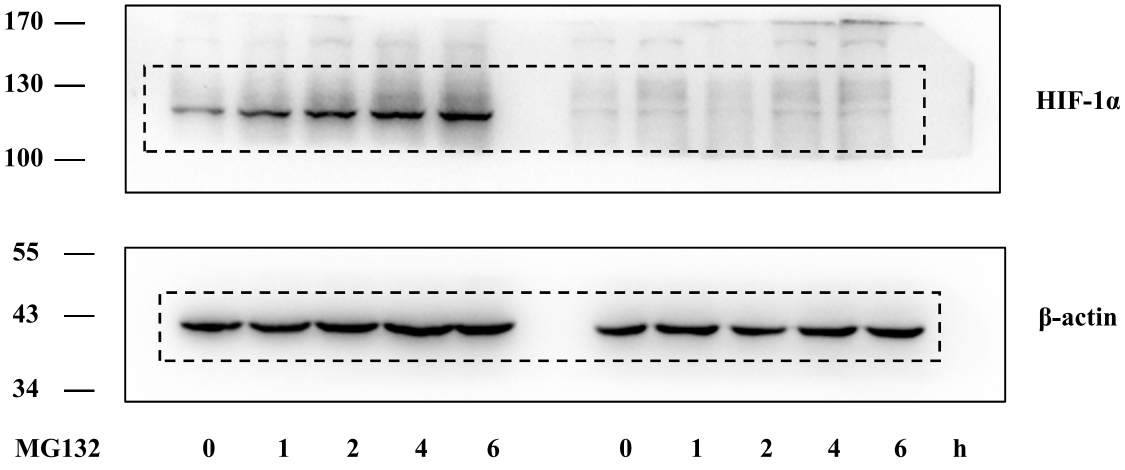

Uncropped Western blotting results in Figure 6b and Figure S6c.
Membranes were often cut to enable blotting with multiple primary antibodies.

Figure S17

Figure 6e

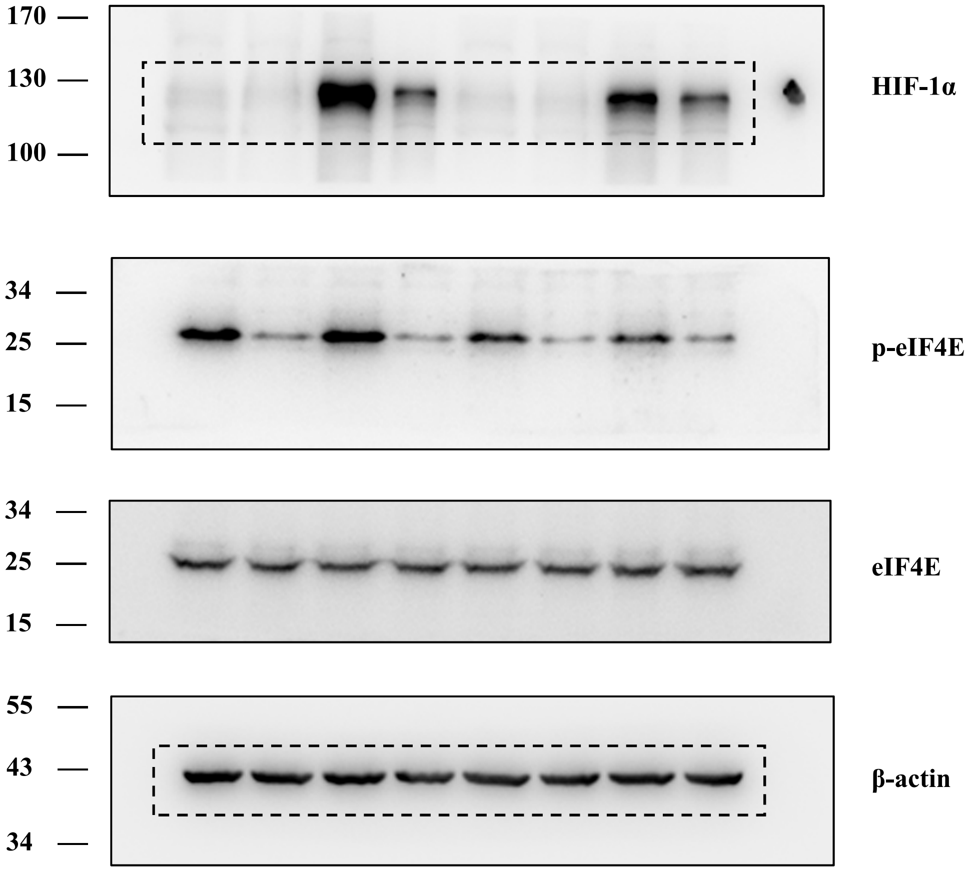

Figure S6b

F0 Vec	F0 OV	F10 WT	F10 KO	A375 WT	A375 KO
1	2	3	4	5	6

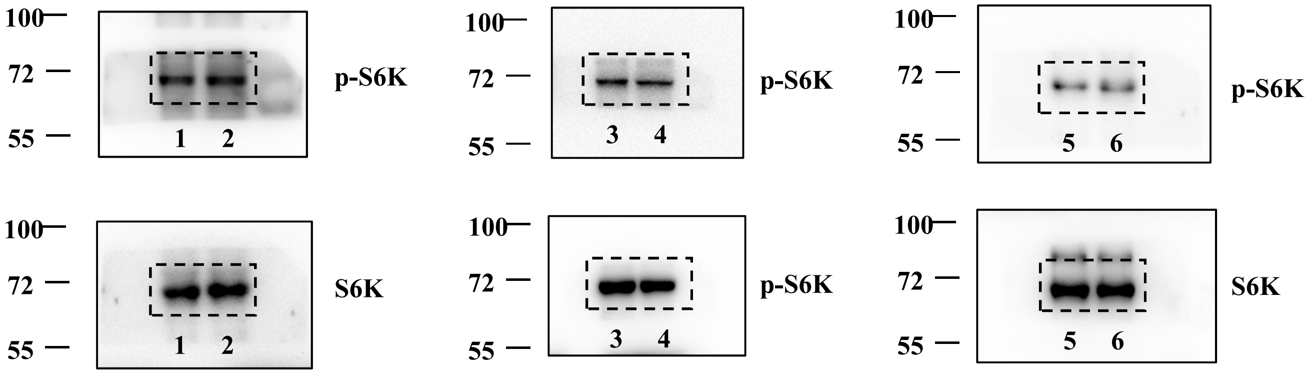

Uncropped Western blotting results in Figure 6e and Figure S6b.
Membranes were often cut to enable blotting with multiple primary antibodies.

Figure S17

Figure S6b

F0 Vec	F0 OV	F10 WT	F10 KO	A375 WT	A375 KO
1	2	3	4	5	6

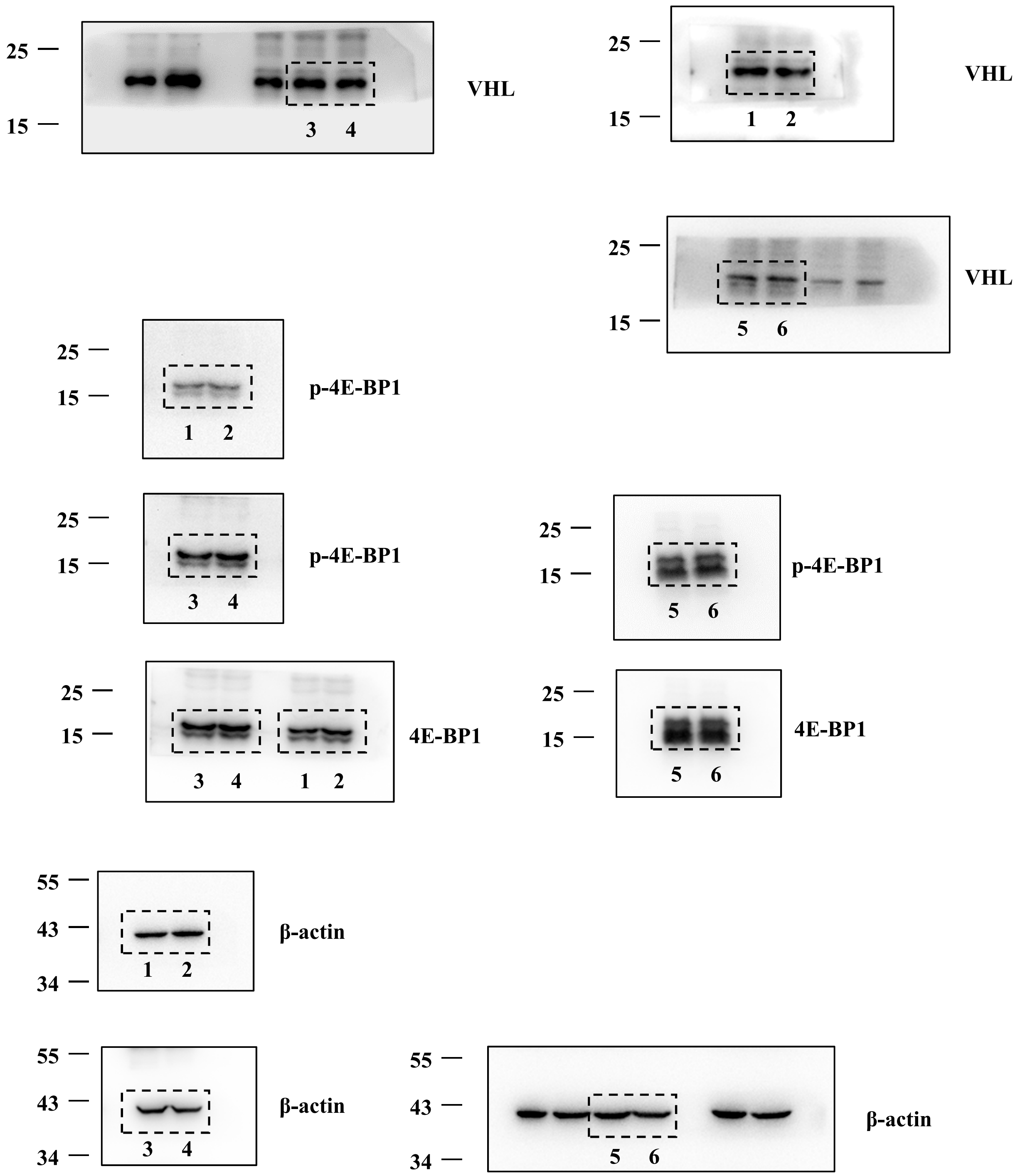

Uncropped Western blotting results in Figure6e and Figure S6b.
Membranes were often cut to enable blotting with multiple primary antibodies.
