# Supplementary material for: CXCR7 promotes melanoma tumorigenesis via Src kinase signaling
Source: Cell Death Dis. 2019 Feb 25;10(3):191. doi: 10.1038/s41419-019-1442-3 (PMC6389959; doi:10.1038/s41419-019-1442-3)
Supplement: Supplementary file 9 — Supplementary table [file 41419_2019_1442_MOESM9_ESM.doc]

**Supplementary Table 1. Sequences of primers for qRT-PCR.**

| Gene symbol | Forward primer (5’ – 3’) | Reverse primer (5’ – 3’) |
| --- | --- | --- |
| *Ccr1* | CTCATGCAGCATAGGAGGCTT | ACATGGCATCACCAAAAATCCA |
| *Ccr2* | GAGCCTGATCCTGCCTCTACTTG | CCTGCATGGCCTGGTCTAAGTGC |
| *Ccr3* | GCTTTGAGACCACACCCTATG | TTCAGGCAATGCTGCCAGTCC |
| *Ccr4* | CCAAAGATGAATGCCACAGAG | CGAACAGCAAATCCGAGATG |
| *Ccr5* | GCTGAAGAGCGTGACTGAT | GAGGACTGCATGTATAATG |
| *Ccr6* | GTGCCAATTGCCTACTCC | GGCTCACAGACATCACGATC |
| *Ccr7* | TTCCAGCTGCCCTACAATGG | GAAGGTTGTGGTGGTCTCCG |
| *Ccr8* | CAGGACCAGAGCCATCAAG | GATGTCATCCAGGGTGGAAG |
| *Ccr9* | GCTGATCTGCTCTTTCTTG | GTGCTTGGATGACTTCTTGG |
| *Ccr10* | GTACGATGAGGAGGCCTATTC | CGTGCGATGGCCACATAG |
| *Cxcr1* | CGTCATGGATGTCTACGTGC | GTAGCAGACCAGCATAGTG |
| *Cxcr2* | ATGCCCTCTATTCTGCCAGAT | GTGCTCCGGTTGTATAAGATGAC |
| *Cxcr3* | GAACGTCAAGTGCTAGATGCCTCG | GTACACGCAGAGCAGTGCG |
| *Cxcr4* | GAAGTGGGGTCTGGAGACTAT | TTGCCGACTATGCCAGTCAAG |
| *Cxcr5* | CGAAGCGGAAACTAGAGCC | CCAGCTTGGTCAGAAGC |
| *Cxcr6* | CAGCTCTGTACGATGGGCAC | CGGTTGAAGGCCTTGGTAGC |
| *Ackr3* | AGCCTGGCAACTACTCTGACA | GAAGCACGTTCTTGTTAGGCA |
| *Cx3cr1* | GACGATTCTGCTGAGGCCTG | GCCCAGACTAATGGTGAC |
| *Xcr1* | CTCAGCCTTGTGGGTAACAGC | ACAGGCAGTAGACAGGAGAAC |
| *HIF1A* | GGCGCGAACGACAAGAAAAAG | CCTTATCAAGATGCGAACTCACA |
| *Hif1a* | ACCTTCATCGGAAACTCCAAAG | ACTGTTAGGCTCAGGTGAACT |
| *Actb* | GGCTGTATTCCCCTCCATCG | CCAGTTGGTAACAATGCCATGT |
| *Ackr1* | GACTACAGCCTGACACCAGC | GTAGCCCAGGTTGCATAGGG |
| *Ackr2* | TTTGCAGGAAGGACGAGGTC | CTCCATCGTCCGTCTTCGAG |
| *Ackr4* | GTCTCTGGAATGCAGTTTCTGGC | GGTATGCTCAGCAAGATGGCAG |

**Supplementary Table 2. Information of antibodies used in Western blot analysis.**

| Antibody | Host species | Supplier | Catalog number | Dilution |
| --- | --- | --- | --- | --- |
| phospho-AKT (Ser 473) | Rabbit | Cell Signaling Technology | 4060 | 1:2000 |
| AKT (total) | Rabbit | Cell Signaling Technology | 9272 | 1:4000 |
| phospho-Src (Tyr 416) | Rabbit | Cell Signaling Technology | 6943 | 1:2000 |
| Src (total) | Rabbit | Cell Signaling Technology | 2123 | 1:4000 |
| phospho-S6K (Thr 389) | Rabbit | Cell Signaling Technology | 9234 | 1:1000 |
| S6K (total) | Rabbit | Cell Signaling Technology | 2708 | 1:3000 |
| phospho-ERK1/2 (Thr 202/Tyr 204) | Rabbit | Cell Signaling Technology | 4377 | 1:2000 |
| ERK1/2 (total) | Rabbit | Cell Signaling Technology | 4695 | 1:4000 |
| phospho-4E-BP1 (Thr 37/46) | Rabbit | Cell Signaling Technology | 2855 | 1:2000 |
| 4E-BP1 (total) | Rabbit | Cell Signaling Technology | 9644 | 1:3000 |
| phospho-eIF4E (Ser 209) | Rabbit | Cell Signaling Technology | 9741 | 1:1000 |
| eIF4E (total) | Rabbit | Cell Signaling Technology | 9742 | 1:2000 |
| β-arrestin2 | Rabbit | Cell Signaling Technology | 3857 | 1:3000 |
| HIF-1α | Mouse | Abcam | ab1 | 1:2000 |
| β-arrestin1 | Rabbit | Abcam | ab32099 | 1:2000 |
| CXCR7 | Rabbit | GeneTex | GTX100027 | 1:2000 |
| EGFR | Mouse | Santa Cruz | sc-120 | 1:1000 |
| VHL | Rabbit | GeneTex | GTX101087 | 1:1000 |
| β-actin | Mouse | GeneTex | GTX629630 | 1:4000 |
| HRP-conjugated anti-rabbit IgG | Goat | Abcam | ab6721 | 1:3000 |
| HRP-conjugated anti-mouse IgG | Goat | Abcam | ab6789 | 1:3000 |

**Supplementary Table 3. The clinical features of patient samples in the tissue microarray.**

| Characteristic |  |  |
| --- | --- | --- |
| Age (years) |  |  |
|  | Median (range) | 51 (0.5-88) |
| Gender |  | Patient number (n) |
|  | Male | 51 |
|  | Female | 49 |
| Clinical stage |  |  |
|  | Benign | 24 |
|  | Malignant-stage I | 1 |
|  | Malignant-stage II | 48 |
|  | Malignant-stage III | 5 |
|  | Malignant-stage IV | 2 |
|  | Metastasis | 20 |

**Supplementary Table 4. Sequences of sgRNAs and primers for CXCR7 deletion.**

| sgRNAs sequences (The PAM sequences are indicated in bold) | | | |
| --- | --- | --- | --- |
| Target | Upstream sgRNA (5’ – 3’) | Downstream sgRNA (5’ – 3’) | Deleted sequence length |
| *Ackr3* | TCGTCATCACCATCCCCGTC **TGG** | GCTTCATCAACCGCAACTAC **AGG** | 669 bp |
| *ACKR3* | GATTGCCCGCCTCAGAACGA **TGG** | GCTTCATCAATCGCAACTAC **AGG** | 965 bp |
| Primers for genomic DNA amplification | | | |
| Target | Forward primer (5’ – 3’) | Reverse primer (5’ – 3’) | Amplicon in wild type cells |
| *Ackr3* | ACTACTCTGACATCAACTGGC | TCTGGAGGCATCAATGAGCTT | 1007 bp |
| *ACKR3* | GGCACACCTTGCTGTTGAAG | GAGAGAATGGATGCAGGGGG | 2057 bp |

**Supplementary Table 5. The targeted sequences of siRNAs.**

| Target | Sense (5’ – 3’) | Antisense (5’ – 3’) |
| --- | --- | --- |
| *Arrb1* | CCUGGUGGAUCCUGAGUAUTT | AUACUCAGGAUCCACCAGGTT |
|  | **CCUUUGAGAUCCCGCCAAATT** | **UUUGGCGGGAUCUCAAAGGTT** |
|  | CCAGCUCAACAUUCUGCAATT | UUGCAGAAUGUUGAGCUGGTT |
| *Arrb2* | GCUGAAGAAGUUGGGCCAATT | UUGGCCCAACUUCUUCAGCTT |
|  | GGAACUCUGUGCGGCUUAUTT | AUAAGCCGCACAGAGUUCCTT |
|  | **CCAUGUCACCAACAAUUCUTT** | **AGAAUUGUUGGUGACAUGGTT** |
| *ARRB2* | GAACAAGAUGACCAGGUAUTT | AUACCUGGUCAUCUUGUUCTT |
|  | **GGAUGACGACUAUGAUGAUTT** | **AUCAUCAUAGUCGUCAUCCTT** |
|  | GCUGUGUUCAUACCUAAAUTT | AUUUAGGUAUGAACACAGCTT |
| *HIF1A* | **CGAGGAAGAACUAUGAACATT** | **UGUUCAUAGUUCUUCCUCGTT** |
|  | GAUGAAAGAAUUACCGAAUTT | AUUCGGUAAUUCUUUCAUCTT |
|  | CUCCCUAUAUCCCAAUGGATT | UCCAUUGGGAUAUAGGGAGTT |

The most efficient siRNAs confirmed by Western blot are shown in bold.
