# Supplementary material for: CXCR7 promotes melanoma tumorigenesis via Src kinase signaling
Source: Cell Death Dis. 2019 Feb 25;10(3):191. doi: 10.1038/s41419-019-1442-3 (PMC6389959; doi:10.1038/s41419-019-1442-3)
Supplement: Supplementary file 10 — Informed Patient Consent [file 41419_2019_1442_MOESM10_ESM.pdf]

Xi'an Alena Biotechnology Ltd., Co.

Tel: 029-89198505, 13324555192

Website: http://www.alenabio.com/

Xi'an Alena Biotechnology Ltd., Co.

September, 2016

To Whom It May Concern:

This is to confirm the legitimacy of tissue resources that are used to make tissue-derived products supplied by Xi'an Alena Biotechnology Ltd., Co.

We hereby certify that all our tissue samples are received from the certified hospitals who guarantee: (1) All the human tissue samples were and will be collected with informed consents from the donors and their relatives, while the documents are not provided to Xi'an Alena Biotechnology Ltd., Co.; (2) All tissue samples were and will be excised by licensed Medical Doctors; (3) All tumor tissue samples were and will be diagnosed and identified by at least two different evaluators.

We follow standard medical care and protect the donors' privacy. The donor's identity is anonymity and tissue microarray samples are not coded in any way that would allow patients to be identified. US Biomax does not releases any patient information with our products either openly or in coded information. If you have more questions please feel free to contact us.

Sincerely,
Xin Chen
Chief Scientific Officer

Xi'an Alena Biotechnology Ltd., Co.
Tel: 029-89198505，029-62221350

CONSENT FOR DONATION OF ANATOMICAL GIFT

Donor Name:____(Print)

I,__________________________, am (the) ● healthcare power of attorney, ● court appointed legal guardian, ● spouse, ● son or daughter eighteen years or older, ● parent, ● domestic partner, ● brother or sister eighteen years or older, ● person/agency, or ● myself authorized by law to handle the affairs of the above named person.
I, in accordance with the Anatomical Gift Act and for humanitarian reasons, hereby grant consent for:

- **Entire body to____________________________, without remains**
  This type of donation may include but is not limited to eyes, skin, bone, internal organs, and all extremities
- **Entire body to the__________________________; with remains for cremation**
  This type of donation may include but is not limited to eyes, skin, bone, internal organs, and all extremities.

- **The following specific organ(s) ortissue(s): ______________________________**

for entities reviewed and approved by____________________________, non-profit and/or commercial, to use aforementioned tissues for medical research, medical education, processing, alteration, isolation, extraction and or redistribution, to be transferred to other countries.
I understand that any additional charges directly associated with the donation will be assumed by______________________. I further understand that there is no guarantee that the donation will take place. I hereby authorize__ to obtain a complete medical history, autopsy findings, and blood samples as necessary to ensure the safety of the organs/tissues and to determine their suitability. Such testing may include, but is not limited to hepatitis, and the AIDS virus. These testresults may be disclosed to the donor's physician of record and may be reported to the appropriate agencies as required by law.

By donating the decedent's entire body *without* remains to________, I understand the remaining tissue and/or organs will not be returned for burial and/or cremation purposes. (initial here, if applicable)

The nature and purpose of the procedure have been explained to me, and I acknowledge that no guarantee or assurance has been made as to the results that may be obtained from the research or study of the specified organs or tissues. This authorization is volunteered without obligation of any kind on the part of the recipient, hospital,________or any individual or any organization authorized by law to receive this donation and is motivated by humanitarian instincts without hope or expectation of reward or compensation of any kind. I release any and all claims that I may have in my capacity as the person/agent authorized to dispose of the donor's tissue received by ________. I understand the contents of this document and hereby authorize the above stated

anatomical gift(s).

Signature of person granting consent:____________________Date:____/____/____Time: ______ AM/PM

Address:________________________________ Witness: ____________________________

________________________________Witness: ____________________________

Telephone Number: ________________________

Next of Kin:______________________________ Next of Kin Telephone: ________________

Next of Kin Address: ____
