# Supplementary material for: CXCR7 promotes melanoma tumorigenesis via Src kinase signaling
Source: Cell Death Dis. 2019 Feb 25;10(3):191. doi: 10.1038/s41419-019-1442-3 (PMC6389959; doi:10.1038/s41419-019-1442-3)
Supplement: Supplementary file 11 — supplemental figure legends [file 41419_2019_1442_MOESM11_ESM.docx]

**Supplementary figure legends**

**Figure S1. The expression of CXCR7 in murine melanoma cells.** (a and b) The expression levels of chemokine receptors in B16-F0 cells (a) and B16-F1 (b) cells were evaluated by qRT-PCR and normalized to *Ccr1* mRNA level. The qRT-PCR experiments were independently repeated three times. Data are presented as mean ± SD.

**Figure S2. CXCR7 facilitates melanoma cell proliferation *in vitro* and tumor growth *in vivo*.** (a) The relative mRNA levels of CXCR7 in F0 Vec and F0 OV cells. The mRNA levels were normalized to F0 cells transfected with control vector. (b) Validation of CXCR7 depletion in B16-F10 cells by amplification of genomic DNA. A lower band in CXCR7-depleted cells revealed a complete deletion of the targeted sequence. The amplicons from the wild type and deleted alleles are 1007 bp and 338 bp, respectively. (c) The secretion of CXCL12 by murine melanoma cells. (d) Immunohistochemistry staining of CXCR7 in tumors derived from mice bearing murine melanoma cells. The staining intensities of CXCR7 were normalized to F0 Vec tumors (top) or F10 WT tumors (bottom). Left: representative images; scale bar = 100 μm. Right: relative quantitative results of staining intensities (n = 6 - 8 for each group). (e) Immunohistochemistry staining of Ki67 in the tumors derived from mice bearing murine melanoma cells. Left: representative images; scale bar = 100 μm. Right: quantitative results of Ki67 positive cells (n = 6 - 8 for each group). (f) Validation of CXCR7 depletion in A375 cells. The amplicon from the wild type and deleted alleles are 2057 bp and 1092 bp, respectively. (g) The protein levels of CXCR7 in A375 WT and KO cells. (h) Immunohistochemistry staining of CXCR7 and Ki67 in A375 WT and KO tumors. The staining intensities of CXCR7 were normalized to A375 WT tumors. Left: representative images; scale bar = 100 μm. Right: quantitative results (n = 6 for each group). (i) The effects of the CXCR7-blocking antibody on A375 tumor growth (n = 6 for each group). A375 WT or KO cells were subcutaneously implanted into nude mice. The mice were intraperitoneally administered with the CXCR7-blocking antibody (1 mg/kg, 331110, Biolegend) or the IgG isotype control (1 mg/kg, 400348, Biolegend) every other day. The qRT-PCR experiments and ELISA assays were independently repeated three times. The quantification of immunohistochemistry staining was evaluated in 12 random fields for each tumor. Tumor volumes are shown as mean ± SEM, and other data are presented as mean ± SD; **p* < 0.05, ***p* < 0.01, ****p* < 0.001; ns, not significant.

**Figure S3. CXCR7 promotes melanoma proliferation through Src activation.** (a and e) CXCL12 treatment activated Src kinase in the presence of AMD3100. F10 WT (a) and A375 WT (e) cells were cultured in serum-free medium overnight. After pretreated with AMD3100 (1 μg/ml) for 1 h, the cells were incubated with recombinant murine or human CXCL12 at indicated concentration. The phosphorylation levels of Src were detected by Western blot. The optimal concentrations of recombinant murine and human CXCL12 were determined as 50 ng/ml and 100 ng/ml, respectively. (b, c, d) Apoptosis measurements by flow cytometry in melanoma cells treated with DMSO or PP1. Indicated cell lines were seeded into 6-well plates in the presence of DMSO or PP1 (10 μM). Cell apoptosis rates were determined by flow cytometry. Left: representative FACS profiles. Right: quantitative results of apoptosis experiments. (f) Western blot analysis of EGFR in murine and human melanoma cells. 4T1 and A549 cells were used as positive controls. (g, h, i) The impacts of the EGFR inhibitor AG1478 on melanoma cell proliferation. The constructed melanoma cells were seeded into 96-well plates in the presence of DMSO or AG1478 (10 μM). After 48 h, the numbers of cells were examined by CCK-8 assays. The proliferation rates were normalized to F0 Vec cells (g), F10 WT cells (h) or A375 WT cells (i). Proliferation experiments and apoptosis assays were independently repeated in triplicate. Data are presented as mean ± SD; ns, not significant.

**Figure S4. CXCR7 stimulates Src kinase phosphorylation through β-arrestin2.** (a) Identification of the siRNAs targeting β-arrestin1 and β-arrestin2 in F10 WT cells. Three siRNAs with different targets were designed to downregulate β-arrestin1 and β-arrestin2 in murine cells. The knockdown efficiencies were confirmed by Western blot. The most efficient siRNA was used in further experiments. (b, c, f) The effects of siRNA transfection on melanoma cell apoptosis. Indicated cell lines transfected with scramble siRNA or β-arrestin2 siRNA were seeded into 6-well plates. Cell apoptosis rates were detected by flow cytometry. Left: representative FACS profiles. Right: quantitative results of apoptosis experiments. (d) Identification of the siRNA targeting β-arrestin2 in A375 WT cells. Three siRNAs with different targets were designed to knockdown β-arrestin2 in human cells. The most efficient siRNA was determined by Western blot and used in further experiments. (e) The effect of β-arrestin2 knockdown on Src kinase phosphorylation in A375 WT cells. Apoptosis assays were independently conducted in triplicate. Data are presented as mean ± SD; ns, not significant.

**Figure S5. CXCR7 contributes to melanoma angiogenesis and promotes VEGF secretion by upregulating HIF-1α expression.** (a) Immunofluorescent staining of CD31 (green) and DAPI (blue) in A375 WT and KO tumors. The blood vessel densities were calculated by dividing CD31 area by DAPI area, and were normalized to A375 WT tumors. Left: representative images; scale bar = 50 μm. Right: relative quantitative results (n = 6 for each group). (b) Immunohistochemistry staining of VEGF and HIF-1α in A375 WT and KO tumors. The staining intensities of VEGF and HIF-1α were normalized to A375 WT tumors. Left: representative images; scale bar = 100 μm. Right: relative quantitative results of staining intensities (n = 6 for each group). (c) The secretion of VEGF by A375 WT and KO cells with or without CoCl_2_ (200 μM) treatment. The cells were pretreated with CoCl_2_ for 6 h, and then incubated in serum-free medium containing CoCl_2_ overnight. The conditioned mediums were collected and the levels of VEGF were determined by ELISA. (d) HIF-1α expression in A375 WT and KO cells treated with CoCl_2_. (e) Identification of siRNA targeting HIF-1α in A375 cells. Three siRNAs with different targets were designed to knockdown HIF-1α in human cells. The cells transfected with indicated siRNAs were treated with or without CoCl_2_ for 6 h. The most efficient siRNA was identified by Western blot. ELISA assays were independently conducted in triplicate. The quantification of immunofluorescent and immunohistochemistry staining were evaluated in 12 random fields for each tumor. Data are presented as mean ± SD; ***p* < 0.01, ****p* < 0.001.

**Figure S6. CXCR7 accelerates HIF-1α translation by facilitating Src-mediated eIF4E phosphorylation.** (a) The impacts of CXCR7 modulations on HIF-1α transcription in melanoma cells. The mRNA levels were normalized to F0 Vec cells (left), F10 WT cells (middle) or A375 WT cells (right). (b) The effects of CXCR7 modulations on the levels of VHL, phosphorylated S6K and phosphorylated 4E-BP1. (c) HIF-1α expression in A375 WT and KO cells treated with MG132 (10 μM). The qRT-PCR experiments were independently conducted three times. Data are presented as mean ± SD; ns, not significant.
